# miRNA‐200c‐3p promotes endothelial to mesenchymal transition and neointimal hyperplasia in artery bypass grafts

**DOI:** 10.1002/path.5574

**Published:** 2020-11-28

**Authors:** Dan Chen, Cheng Zhang, Jiangyong Chen, Mei Yang, Tayyab A Afzal, Weiwei An, Eithne M Maguire, Shiping He, Jun Luo, Xiaowen Wang, Yu Zhao, Qingchen Wu, Qingzhong Xiao

**Affiliations:** ^1^ Department of Cardiothoracic Surgery The First Affiliated Hospital of Chongqing Medical University Chongqing PR China; ^2^ Centre for Clinical Pharmacology, William Harvey Research Institute Barts and The London School of Medicine and Dentistry, Queen Mary University of London London UK; ^3^ Department of Cardiothoracic Surgery Yongchuan Hospital of Chongqing Medical University Chongqing PR China; ^4^ Vascular Surgery The First Affiliated Hospital of Chongqing Medical University Chongqing PR China; ^5^ Key Laboratory of Cardiovascular Diseases at The Second Affiliated Hospital School of Basic Medical Sciences, Guangzhou Medical University Guangzhou PR China; ^6^ Guangzhou Municipal and Guangdong Provincial Key Laboratory of Protein Modification and Degradation School of Basic Medical Sciences, Guangzhou Medical University Guangzhou PR China

**Keywords:** miRNA‐200c‐3p, endothelial to mesenchymal transition, neointima, arterial bypass graft, post‐angioplasty restenosis, atherosclerosis, microRNA, endothelial cell

## Abstract

Increasing evidence has suggested a critical role for endothelial‐to‐mesenchymal transition (EndoMT) in a variety of pathological conditions. MicroRNA‐200c‐3p (miR‐200c‐3p) has been implicated in epithelial‐to‐mesenchymal transition. However, the functional role of miR‐200c‐3p in EndoMT and neointimal hyperplasia in artery bypass grafts remains largely unknown. Here we demonstrated a critical role for miR‐200c‐3p in EndoMT. Proteomics and luciferase activity assays revealed that fermitin family member 2 (*FERM2*) is the functional target of miR‐200c‐3p during EndoMT. *FERMT2* gene inactivation recapitulates the effect of miR‐200c‐3p overexpression on EndoMT, and the inhibitory effect of miR‐200c‐3p inhibition on EndoMT was reversed by *FERMT2* knockdown. Further mechanistic studies revealed that FERM2 suppresses smooth muscle gene expression by preventing serum response factor nuclear translocation and preventing endothelial mRNA decay by interacting with Y‐box binding protein 1. In a model of aortic grafting using endothelial lineage tracing, we observed that miR‐200c‐3p expression was dramatically up‐regulated, and that EndoMT contributed to neointimal hyperplasia in grafted arteries. MiR‐200c‐3p inhibition in grafted arteries significantly up‐regulated FERM2 gene expression, thereby preventing EndoMT and reducing neointimal formation. Importantly, we found a high level of EndoMT in human femoral arteries with atherosclerotic lesions, and that miR‐200c‐3p expression was significantly increased, while *FERMT2* expression levels were dramatically decreased in diseased human arteries. Collectively, we have documented an unexpected role for miR‐200c‐3p in EndoMT and neointimal hyperplasia in grafted arteries. Our findings offer a novel therapeutic opportunity for treating vascular diseases by specifically targeting the miR‐200c‐3p/FERM2 regulatory axis. © 2020 The Authors. *The Journal of Pathology* published by John Wiley & Sons, Ltd. on behalf of The Pathological Society of Great Britain and Ireland.

## Introduction

Although coronary artery bypass grafting (CABG) is one of the most successful procedures for treating patients with coronary artery disease (CAD), and has been recommended as the gold standard for patients with multiple‐vessel disease [1, 2] and/or left main CAD regardless of the patient's SYNTAX score (low, intermediate or high) [3, 4, 5], long‐term survival rates in these patients are still poor and strongly limited by the development of graft vasculopathy or failure due to neointimal lesion formation. Neointimal smooth muscle cell (SMC) hyperplasia is the key pathophysiological mechanism of vascular diseases including vascular graft restenosis [6]. Despite decades of investigations, the origin of neointimal cells remains controversial, with increasing evidence pinpointing a contribution of medial SMCs, stem/progenitor cells [7, 8], and endothelial–mesenchymal transition (EndoMT) [9, 10, 11, 12, 13]. EndoMT is a transition process characterized by loss of cell–cell adhesions and changes in cell polarity, with reduced expression of endothelial cell (EC) markers but increased expression of mesenchymal cell (or SMC‐like) markers. The resultant cells acquire myofibroblast‐like characteristics with contractile function, enhanced migratory and proliferative phenotype, and increased extracellular matrix production. Meanwhile, they lose EC functional characteristics with an impaired anti‐thrombogenicity and angiogenesis [11, 14, 15].

Accumulating evidence from preclinical studies and histological observations in humans has provided clear evidence that EndoMT not only plays a fundamental role in normal embryonic developmental processes [14, 15, 16] but also contributes to a variety of pathological conditions including ventricular diastolic dysfunction [17], atherosclerosis [13, 18] and atherosclerotic plaque instability [10], pulmonary arterial hypertension [9], vascular graft remodelling [12], vascular and valvular calcifications [19, 20], and tumour progression, among others [15, 16, 21, 22, 23, 24]. Therefore, exploring the underlying molecular mechanisms of EndoMT is key to developing novel therapeutic strategies aimed at treating these conditions. Although multiple intercellular mechanisms and signal pathways have been suggested to govern EndoMT [15, 16, 18, 21, 22, 25, 26], the exact molecular mechanisms involved in cardiovascular pathogenesis that occur as a result of EndoMT remain obscure.

MicroRNAs (miRNAs) are short (20–23 nt) and conserved non‐coding RNAs with profound roles in cardiovascular diseases. A handful of miRNAs have been reported to play a role in regulating EndoMT by targeting one or multiple EndoMT‐associated genes [15, 16, 21, 26, 27]. miRNA‐200c‐3p (miR‐200c‐3p) belongs to the miR‐200 miRNA cluster. The miR‐200 family is overwhelmingly linked with epithelial–mesenchymal transition (EMT) and its inverse processes [28]. Later studies also support the involvement of this miRNA family in EndoMT [16]. Since controversial and cellular context‐dependent roles for the miR‐200 family in EMT or EndoMT have been widely documented [16, 29], more studies are needed to focus on individual members of the miR‐200 family to elucidate their specific role in these pathological conditions. In particular, miR‐200c‐3p has been widely implicated in cardiovascular development and diseases [30]. Importantly, we have demonstrated previously a critical role for miR‐200c‐3p in EC differentiation from human embryonic stem cells [31]. However, the functional implication of this miRNA in EndoMT and vascular grafting‐induced neointimal SMC hyperplasia remains elusive. In the current study, we demonstrate that miR‐200c‐3p promotes EndoMT by partially targeting fermitin family member 2 (*FERM2*), and therefore, modulation of the miR‐200c‐3p/FERM2 regulatory axis in vascular grafts represents a novel therapeutic approach for preventing vascular graft failure in patients with CABG.

## Materials and methods

### Animals and mouse experiments

*Cdh5*‐CreERT2 × Rosa26‐tdTomato mice were generated by crossing Tg(*Cdh5*‐CreERT2) with Rosa26‐CAG‐LSL‐cas9‐tdTomato mice (both are on a C57BL/6J background and were obtained from Gempharmatech, Jiangsu, PR China). Tamoxifen (T5648; Merck, Haverhill, UK) was dissolved in corn oil and administered by gavage (0.15 mg/g body weight, four times) to male *Cdh5*‐CreERT2 × Rosa26‐tdTomato mice to induce Cre recombinase activity and tdTomato expression as described in previous studies [8, 32]. After a 2‐week washout, thoracic aortic segments were harvested and randomly allocated to different experimental groups. Animal husbandry and all experimental procedures were approved and performed in accordance with the guidelines of the Institutional Animal Care and Use Committee of The First Affiliated Hospital of Chongqing Medical University or Queen Mary University of London (PB508B78D). In addition, the principles governing the care and treatment of animals, as stated in the *Guide for the Care and Use of Laboratory Animals* published by the National Academy of Sciences (8th edn, 2011), were followed at all times during this study. All mice were euthanized by placing them under deep anaesthesia with 100% O_2_–5% isoflurane, followed by cervical dislocation.

Mouse aortic isograft transplantation was performed as described previously [33, 34]. The procedures for local miR‐200c‐3p inhibition in the grafted aortas were similar to that described in our previous study [35] with some modifications. In brief, immediately after harvest, 50–60 μl of DMEM containing vehicle (mock transfection, sham), control scrambled locked nucleic acid (LNA) modified oligonucleotides (LNA‐SCR), or LNA‐miR‐200c‐3p per vessel was randomly injected into the arteries, followed by a 30‐min incubation for local transfection of endothelium. After that, aortic segments were transplanted into the carotid artery using end‐to‐end anastomosis.

#### Statistical analysis

Each experiment was performed in at least five biological replicates, and all values are expressed as mean ± standard error of the mean (SEM). Statistical analysis as specified in the figure legends and preparation of plots was performed using GraphPad Prism 8 (GraphPad Inc, San Diego, CA, USA). In brief, the Kolmogorov–Smirnov (K–S) normality test was used for checking the normality of the data. Two‐tailed unpaired Student's *t*‐test was used for comparisons between two groups, or one (or two)‐way analysis of variance (ANOVA) with a Tukey's *post hoc* test was applied when more than two groups were compared. *p* < 0.05 was considered statistically significant.

Details are available in Supplementary materials and methods for Antibodies used, Cell culture and induction of EndoMT, MiR‐200c‐3p inhibitor transfection, Human *FERMT2* 3’‐UTR clone and miR‐200c‐3p binding sites mutation, Human SMC gene reporter plasmids and related SRF binding site mutants, Transient transfection and luciferase activity assays, FERMT2 shRNA lentivirus, Generation of *miR‐200c‐3p* overexpression pseudo‐viral particles, Cell proliferation (CCK‐8) assays, Transwell migration assays, Tube formation, Western blotting, Proteomics studies and data analysis, Reverse transcription–quantitative real‐time PCR (RT‐qPCR), miProfile™ Custom miRNA qPCR Arrays analysis, Chromatin immunoprecipitation (ChIP) assays, RNA immunoprecipitation (RIP) assays, Aortic grafting experiments, Morphometric analysis and quantification of lesion formation, Immunofluorescence analysis, Aortic cell sorting, and Proximity ligation assays (PLAs).

## Results

### miRNA expression during EndoMT

**Figure 1:**
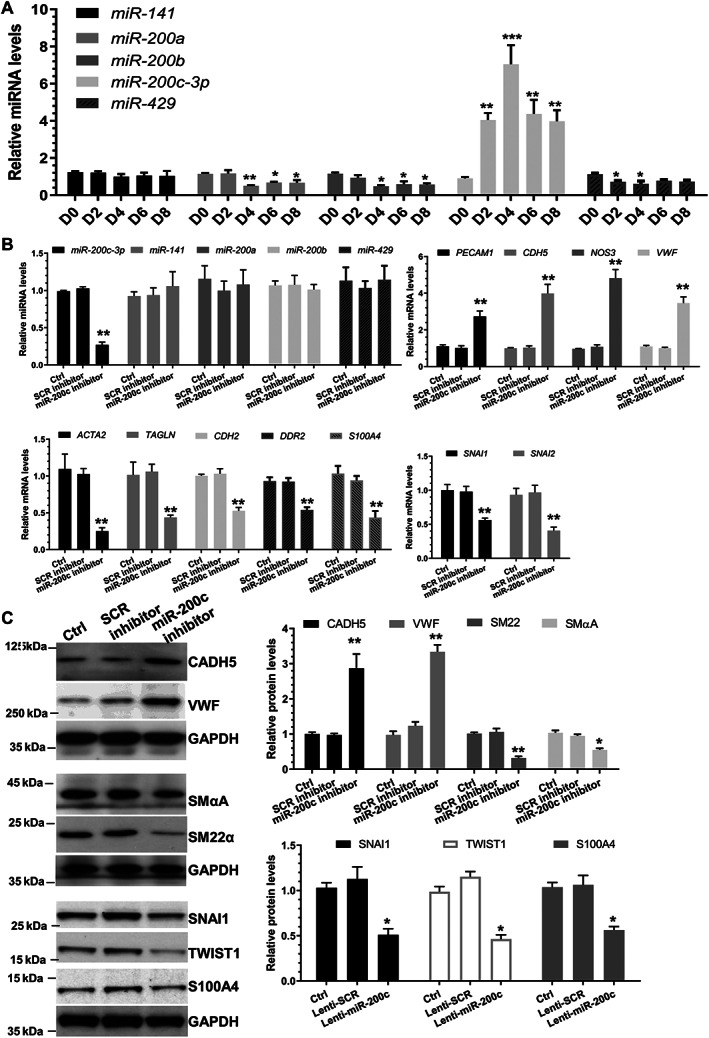
miR‐200c‐3p inhibition prevents EndoMT. (A) Expression levels of the miR‐200 family during EndoMT. HUVECs were incubated with 5 ng/ml TGFβ1 and 5 ng/ml TNFα for the indicated times to induce EndoMT. (B, C) Inhibition of miR‐200c‐3p prevents EndoMT. HUVECs were transfected with reagent control only (Ctrl), a scrambled negative control miRNA inhibitor (SCR inhibitor) or miR‐200c‐3p inhibitor (miR‐200c inhibitor), respectively. Transfected cells were incubated with 5 ng/ml TGFβ1/TNFα for 4 days. Total RNAs and proteins were harvested and subjected to RT‐qPCR (B) and western blot (C) analysis, respectively. Left panel in C: representative images; right panel: quantitative results of five independent experiments. The data presented here are mean ± SEM of five (*n* = 5) independent experiments. *p < 0.05, **p < 0.01, or ***p < 0.001 (versus D0 or Ctrl/SCR; one‐way ANOVA with a Tukey's *post hoc* test).

Human umbilical vein endothelial cells (HUVECs) or aortic endothelial cells (HAoECs) could be stimulated to undergo EndoMT by treating them with a cytokine combination of transforming growth factor‐β1 (TGFβ1) and interleukin‐1β [36] or tumour necrosis factor alpha (TNFα) [37]. HUVECs treated with these two cytokines gradually underwent a clear morphological transition, adopting a more SMC or mesenchymal appearance over the 8‐day treatment (supplementary material, Figure S1A). Coincident with this morphological transition, HUVECs treated with TGFβ1/TNFα displayed a significantly decreased expression of EC markers (*PECAM1*, *CDH5*, *NOS3*, *VWF*, and *KDR*) (supplementary material, Figure S1B), but a dramatically increased expression of mesenchymal/SMC markers (SMαA/*ACTA2*, SM22α/*TAGLN*, *SMTN*, *CDH2*, *DDR2*, and FSP1/*S100A4*) (supplementary material, Figure S1C) and a host of EndoMT‐associated regulators (*SNAI1*, *SNAI2*, *TWIST1*, and *TWIST2*) (supplementary material, Figure S1D). Such changes were further confirmed at protein levels (supplementary material, Figure S1E,F). These data collectively confirmed that TGFβ1/TNFα combinational treatment could induce EndoMT. A customized miRNA RT‐qPCR Array analysis (GeneCopoeia, Rockville, MD, USA) was conducted to identify potential miRNA candidates governing the EndoMT process. Nineteen and 33 miRNAs were significantly up‐ and down‐regulated during EndoMT, respectively (supplementary material, Figure S2). Interestingly, we found that three of the miR‐200 miRNA family members (miR‐200a, ‐200b, and ‐429) were significantly down‐regulated during EndoMT, but the opposite was observed with miR‐200c‐3p expression (supplementary material, Figure S2). This finding was further confirmed using RT‐qPCR analysis (Figure 1A).

### Effect of miR‐200c‐3p on EndoMT

To study the potential effect of miR‐200c‐3p on EndoMT, miR‐200c‐3p loss/gain‐of‐function experiments were conducted in HUVECs undergoing EndoMT. Data from RT‐qPCR analyses confirmed that a panel of EC and mesenchymal/SMC genes were significantly up‐regulated and down‐regulated by miR‐200c‐3p inhibition, respectively (Figure 1B). A similar inhibitory effect was observed with EndoMT‐associated mediators (Figure 1B). Western blot assays showed that miR‐200c‐3p knockdown increased EC marker proteins, but decreased SMC/mesenchymal marker and EndoMT regulatory proteins (Figure 1C). Functionally, miR‐200c‐3p inhibition in HUVECs undergoing EndoMT resulted in a cellular morphological appearance resembling normal ECs (supplementary material, Figure S3A), decreased cell proliferation (supplementary material, Figure S3B) and migration (supplementary material, Figure S3C), and increased angiogenesis (supplementary material, Figure S3D,E). Conversely, the opposite effects were observed when miR‐200c‐3p was overexpressed in HUVECs undergoing EndoMT (supplementary material, Figures S4 and S5). Importantly, a similar role for miR‐200c‐3p in EndoMT was also observed in HAoECs (supplementary material, Figure S6).

### Proteomics analysis to uncover the potential target genes of miR‐200c during EndoMT

Label‐free quantitative proteomics analyses were conducted to search for the potential target genes of miR‐200c‐3p during EndoMT. As shown in supplementary material, Figure S7A, 87 proteins were found to be significantly modulated by miR‐200c‐3p overexpression. Since promoting RNA decay and controlling target gene translational repression is the fundamental regulatory mechanism for most of the miRNAs, the proteins down‐regulated by miR‐200c‐3p overexpression represent possible target genes of miR‐200c‐3p in EndoMT. Interestingly, GO term enrichment analysis of the proteins regulated by miR‐200c‐3p during EndoMT showed that E‐box/cadherin binding, morphogenesis/development of aortic and heart valve and endocardial cushion, mesenchyme morphogenesis, cardiovascular system development and angiogenesis, cellular development, cell migration/adhesion, cell–cell junction organization/assembly, and regulation of metabolic processes were the highly enriched biological processes and/or molecular functions (supplementary material, Figure S7B,C). These observations further supported a role for miR‐200c‐3p in EndoMT. Importantly, by using several computational algorithmic databases, we have identified one or more miR‐200c‐3p binding sites within the 3’‐UTR of 24 (out of 41) genes whose protein expression levels were down‐regulated by miR‐200c‐3p overexpression. As such, these proteins likely represent good candidates as functional direct target genes of miR‐200c‐3p in EndoMT.

### FERM2 is a novel target of miR‐200c‐3p in EndoMT

**Figure 2:**
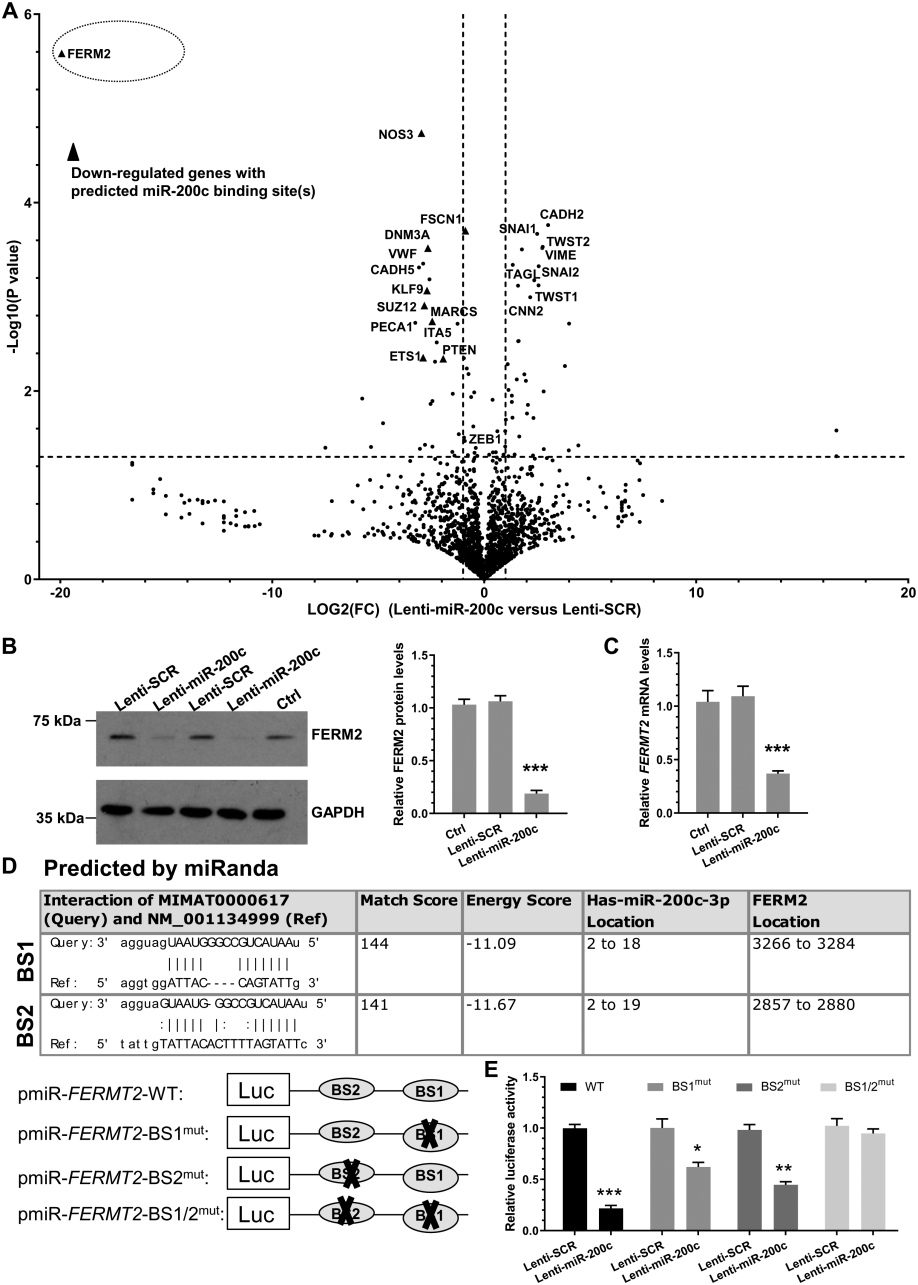
FERM2 was identified as a target gene of miR‐200c‐3p during EndoMT. (A–C) FERM2 expression was down‐regulated by miR‐200c‐3p overexpression. HUVECs were infected with medium control (Ctrl), a scrambled negative control miRNA (Lenti‐SCR) or miR‐200c‐3p overexpression (Lenti‐miR‐200c) lentivirus, respectively. Infected cells were incubated with 5 ng/ml TGFβ1/TNFα for 4 days. Total RNAs and proteins were harvested and subjected to label‐free quantitative proteomics (A), western blot (B), and RT‐qPCR (C) analysis, respectively. Volcano plot in A showing the *P* values (−log_10_) against the fold‐changes of protein expression levels (log_2_) in cells infected with Lenti‐miR‐200c‐3p versus Lenti‐SCR. Triangles indicate the down‐regulated proteins with their mRNA 3’‐UTR containing one or more miR‐200c‐3p binding sites(s). The data presented here are representative images (left panel in B) or mean ± SEM of six (*n* = 6) independent experiments. ***p < 0.001 (versus Ctrl/SCR; one‐way ANOVA with a Tukey's *post hoc* test). (D) Two potential wild‐type binding sites (BS1 and BS2) of miR‐200c‐3p within *FERMT2* 3’‐UTR as predicted by miRanda and their mutants (BS1/2^mut^) are depicted in this illustration. (E) Both binding sites are required for FERM2 repression by miR‐200c‐3p. HUVECs infected with Lenti‐SCR or Lenti‐miR‐200c were transfected with wild‐type *FERMT2* 3’‐UTR reporter (WT), or the indicated single/combined binding site mutants [bindings site 1 (BS1^mut^), 2 (BS2^mut^), or the combinational mutations (BS1/2^mut^)], respectively. Luciferase activity assay was conducted at 48 h post‐transfection. The data presented here are mean ± SEM of five independent experiments (*n* = 5). *p < 0.05, **p < 0.01, ***p < 0.001 (versus SCR, Student's *t*‐test).

As shown in a volcano plot (Figure 2A), fermitin family member 2 (*FERM2* or *FERMT2*) has been clearly signalled out as the most meaningful and important target gene of *miR‐200c‐3p* in EndoMT. Data from western blotting (Figure 2B) and RT‐qPCR (Figure 2C) analyses further confirmed the proteomics data that *miR‐200c‐3p* overexpression significantly decreased FERM2 protein as well as mRNA expression levels. There are two conserved miR‐200c‐3p binding sites within the 3'‐UTR of *FERMT2*, as predicted using the computational algorithm miRanda (Figure 2D). Luciferase assays showed that the luciferase activity of the construct harbouring the wild‐type *FERMT2* 3'‐UTR was dramatically decreased by miR‐200c‐3p overexpression (Figure 2E). Importantly, luciferase activity assays with miR‐200c‐3p binding site single (BS1^mut^or BS2^mut^) or combinational (BS1/2^mut^) mutant reporters demonstrated that both binding sites are required for *FERMT2* gene repression mediated by miR‐200c‐3p (Figure 2E). Collectively, these data confirm that *FERMT2* is a true mRNA target of miR‐200c‐3p in EndoMT.

### *FERMT2* gene suppression is required for miR‐200c‐3p‐mediated EndoMT

To explore the mechanistic link between miR‐200c‐3p and *FERM2* in EndoMT, co‐transduction experiments as indicated in the figures were conducted in HUVECs undergoing EndoMT. RT‐qPCR data showed that the expression level of *FERMT2* was significantly increased by miR‐200c‐3p inhibition, but such induction was abolished by *FERMT2* knockdown in the presence of the miR‐200c‐3p inhibitor (supplementary material, Figure S8A). However, miR‐200c‐3p expression was not regulated by *FERMT2* gene inactivation (supplementary material, Figure S8A), further confirming that the *FERMT2* gene is a downstream target of miR‐200c‐3p. Importantly, RT‐qPCR data showed that miR‐200c‐3p inhibition and *FERMT2* knockdown activated and inhibited EC marker gene expression, respectively, and that EC gene activation by miR‐200c‐3p inhibition was blunted by *FERMT2* knockdown (supplementary material, Figure S8B). The opposite effect was observed with mesenchymal/SMC genes in response to the same treatment (supplementary material, Figure S8C).

### FERM2 regulates SMC gene expression through a transcriptional repression mechanism

**Figure 3:**
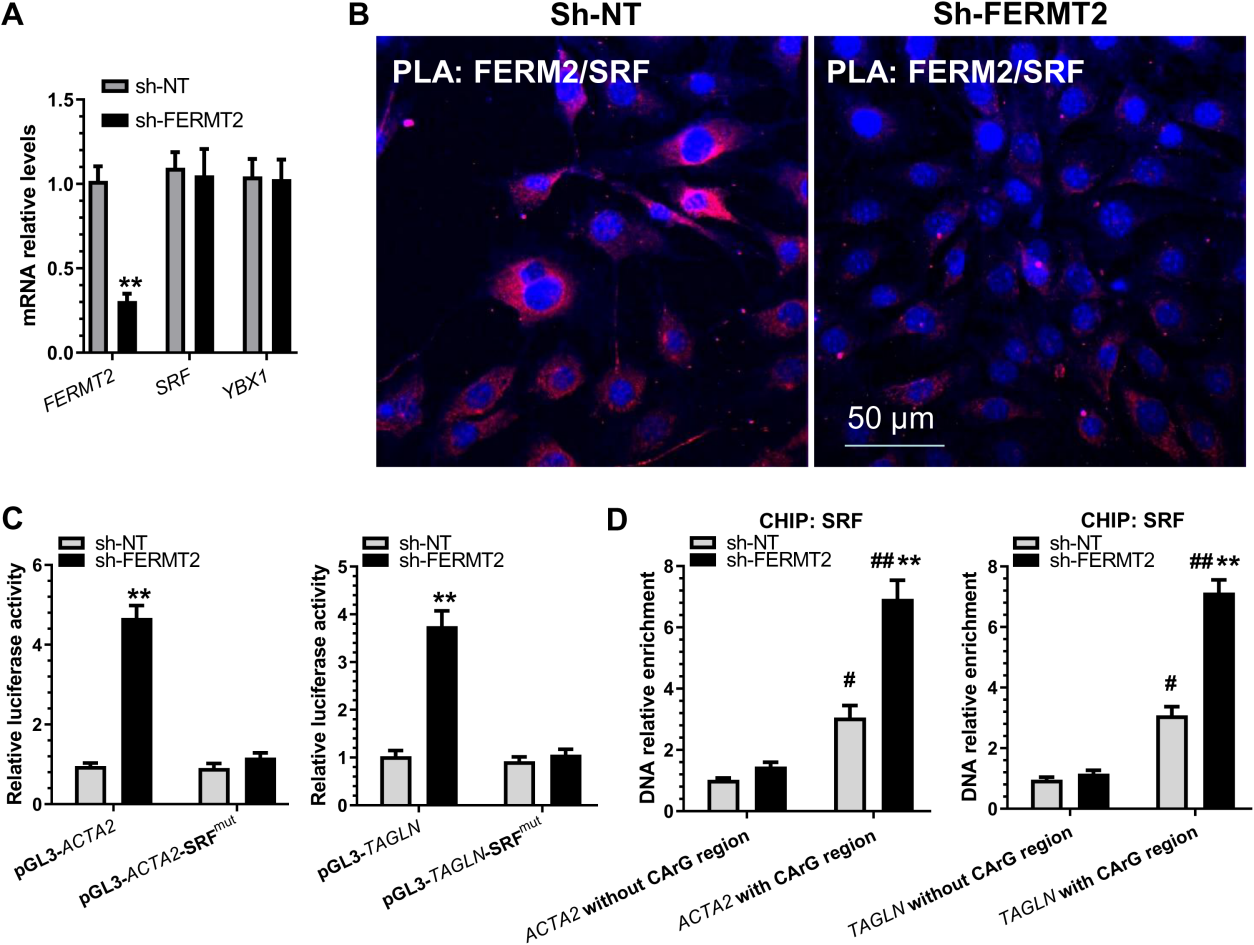
FERM2 suppresses SMC gene expression by interacting with and retaining SRF within cytoplasm. HUVECs infected with a non‐target (sh‐NT) or *FERMT2* gene‐specific shRNA (sh‐FERMT2) lentivirus were incubated with 5 ng/ml TGFβ1/TNFα for 4 days to induce EndoMT. (A) RT‐qPCR analysis of gene expression. (B) Proximity ligation assays (PLAs) using a pair of primary antibodies (or respective IgG controls) as indicated to detect the *in situ* protein interactions of FERM2 with SRF in HUVECs undergoing EndoMT. (C) SMC gene promoter activity assays showed that SRF binding sites(s) are required for FERM2‐mediated SMC gene suppression. HUVECs infected with sh‐NT or sh‐FERM2 lentivirus were transfected with wild‐type SMC gene promoters (pGL3‐*ACTA2*/*TAGLN*) or their SRF binding site mutants (pGL3‐A*CTA2*/*TAGLN*‐SRF^mut^), respectively. Luciferase activity assay was conducted at 48 h post‐transfection. (D) *FERMT2* knockdown increased the binding and enrichment of SRF to SMC gene promoters. ChIP assays were performed using antibody against SRF or normal IgG, respectively, as described in the Materials and methods section. Quantitative PCR amplifications of the adjacent regions without SRF binding sites (CArG element) were included as an additional control for specific promoter DNA enrichment. The data presented here are representative images (B) or mean ± SEM (A, C, D) of five (*n* = 5) independent experiments. **p < 0.05 (versus sh‐NT); ^#^ *p* < 0.05, ^##^ *p* < 0.01 (versus promoter DNA without CArG region). Student's *t*‐test (A) or two‐way ANOVA with a Tukey's *post hoc* test (C, D) was used for statistical analysis, respectively.

Since our data show that both SMC genes (*ACTA2* and *TAGLN*) were activated by *FERMT2* knockdown (supplementary material, Figure S8C), we decided to investigate how SMC gene expression was inhibited by FERM2 during EndoMT. Data retrieved from multiple protein–protein interaction (PPI) databases (e.g. BioGrid, CORUM, IntAct, MINT, and/or STRING) showed a very complicated FERM2 PPI network (supplementary material, Figure S9). Among them, serum response factor (SRF) represents a very interesting candidate protein as SRF is a well‐known master transcriptional factor controlling the expression of a host of SMC genes. We found that the gene expression level of *SRF* was not regulated by *FERMT2* knockdown in HUVECs undergoing EndoMT (Figure 3A). Double immunofluorescence staining showed that while SRF was mainly co‐localized with FERM2 within the cytoplasm of control cells, *FERMT2* knockdown (supplementary material, Figure S10A) or miR‐200c‐3p overexpression (supplementary material, Figure S10B) promoted SRF accumulation within the nuclei. Importantly, the PLA results revealed *in situ* interactions in control cells between FERM2 and SRF within the cytoplasm, while such interactions were dramatically decreased in cells with *FERMT2* knockdown (Figure 3B). Moreover, luciferase activity assays showed that the promoter activity of both SMC genes (*ACTA2* and *TAGLN*) was significantly increased by *FERMT2* knockdown, while such induction disappeared once the SRF binding elements were mutated in these reporters (Figure 3C). Furthermore, ChIP assays confirmed the direct binding of SRF and SMC gene promoters, which was further enhanced by *FERMT2* knockdown in HUVECs undergoing EndoMT (Figure 3D). Collectively, the above data demonstrate that FERM2 suppresses SMC gene expression by preventing SRF nuclear translocation.

### FERM2 regulates EC gene expression through a post‐transcriptional mechanism

**Figure 4:**
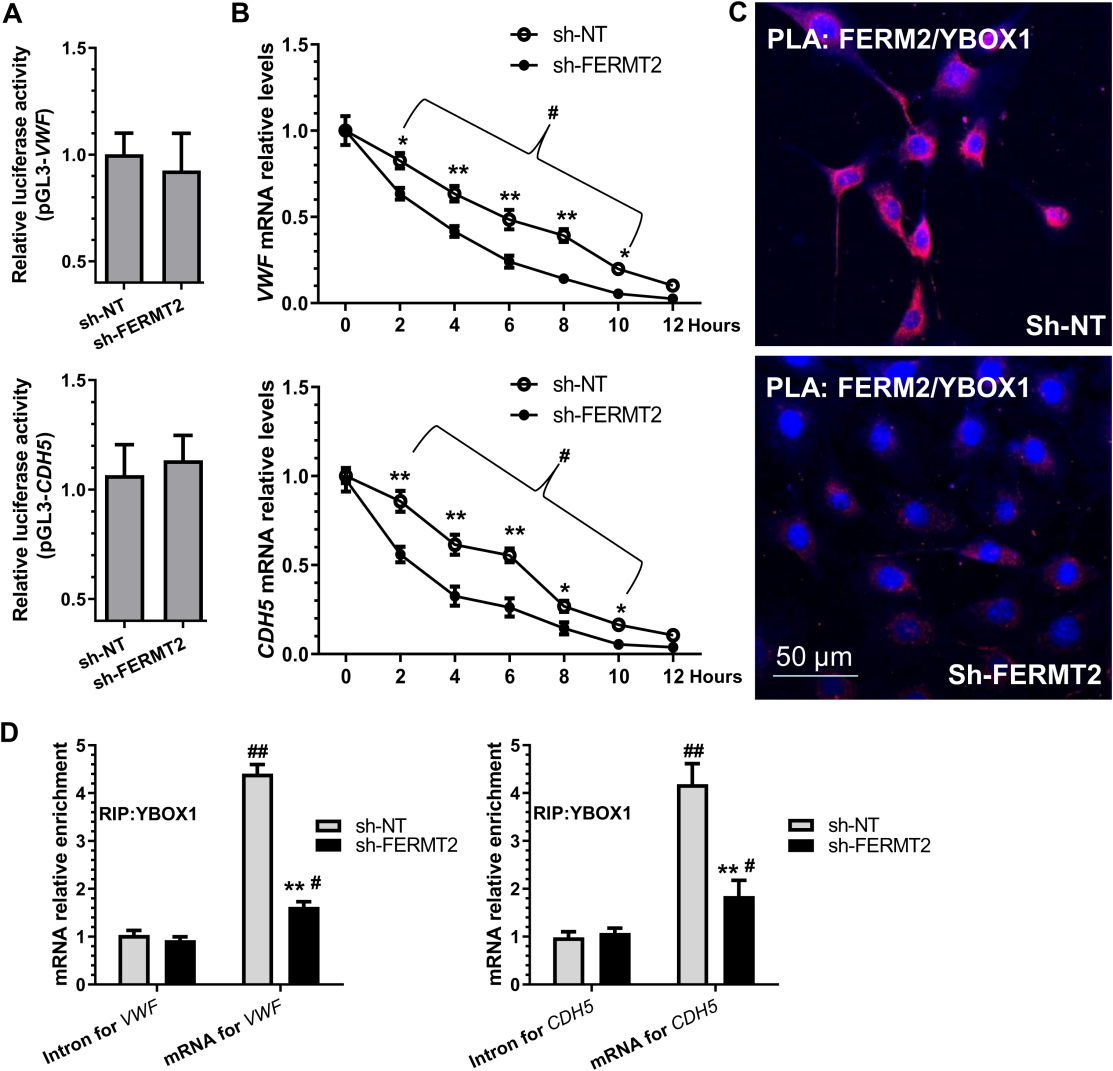
FERM2 regulates EC gene expression by preventing their mRNA decay via interacting with DNA/RNA binding protein YBX1. (A) *VWF* and *CDH5* gene promoter activity was not regulated by FERM2. (B) *VWF* and *CDH5* mRNA degradation was increased by *FERMT2* inhibition. HUVECs infected with a non‐target (sh‐NT) or *FERMT2* gene‐specific shRNA (sh‐FERM2) lentivirus were treated with the transcription inhibitor (actinomycin D, ActD, 1 μg/ml) for the indicated times. The *VWF*/*CDH5* mRNA arbitrary unit at 0 h for both groups was set as 1.0, and at other time points was calculated accordingly. (C) Proximity ligation assays (PLAs) showed the *in situ* protein interactions of FERM2 with YBOX1 in HUVECs. (D) RNA immunoprecipitation (RIP) assays were conducted in HUVECs infected with sh‐NT or sh‐FERM2 lentivirus using antibody against YBOX1 or normal IgG, respectively. Immunoprecipitated RNAs were subjected to RT‐qPCR with the indicated gene primers. The data presented here are representative images (C) or mean ± SEM (A, B, D) of five (*n* = 5) independent experiments. *p < 0.05, **p < 0.01 (versus sh‐NT); ^#^ *p* < 0.05, ^##^ *p* < 0.01 (versus 0 h or Intron). Two‐way ANOVA with a Tukey's *post hoc* test (B) or Student's *t*‐test (D) was used for statistical analysis, respectively.

Conversely, we found that all the EC genes examined in this study were inhibited by *FERMT2* knockdown (supplementary material, Figure S8B). We wondered if EC gene expression was transcriptionally regulated by FERM2. Luciferase activity assays with EC gene (*VWF* and *CDH5*) promoter reporters showed that the EC gene promoter activity was not regulated by *FERMT2* inhibition (Figure 4A), indicating that transcriptional regulation is not behind EC gene regulation by FERM2. Our mRNA stability assays with the transcription inhibitor actinomycin D revealed that *FERMT2* knockdown promoted both *VWF* and *CDH5* mRNA degradation (Figure 4B). To further explore the mechanistic link between FERM2 and EC gene mRNA stability, we re‐scrutinized the FERM2 PPI network (supplementary material, Figure S9) and found that there is potential interaction between FERM2 and Y box binding protein 1 (YBOX1). Among many other important functions, YBOX1 has been specifically recognized as one of the major partners of mRNAs in the cytoplasm and a key mRNA stabilizer [38]. We wondered if FERM2 promotes mRNA stabilization of EC genes through YBOX1. Similar to SRF, we found no evidence to support the notion that FERM2 regulates *YBX1* gene expression (Figure 3A). Proteomics analysis data also showed that the YBOX1 protein level was not significantly affected by miR‐200c‐3p overexpression (fold‐change = 1.21, *p* = 0.628). Instead, data from double immunofluorescence staining (supplementary material, Figure S11) and PLA assays (Figure 4C) confirmed the direct interaction between FERM2 and YBOX1, mainly within the cytoplasm. Importantly, RIP assays showed the direct binding of YBOX1 protein to *VWF* and *CDH5* mRNAs, and such binding was significantly inhibited by *FERMT2* knockdown (Figure 4D). Hence, these data reveal that FERM2 increases EC mRNA stability by working in concert with YBOX1.

### Arterial inhibition of miR‐200c‐3p decreases neointimal hyperplasia in grafted aortas

As mentioned previously, EndoMT is one of the major contributors to vascular graft stenosis [12]. To investigate whether miR‐200c‐3p plays a role in EndoMT in the context of vascular graft remodelling, a well‐established aortic isograft transplantation model [33, 34] was carried out between female C57BL/6J and male cadherin 5 (*Cdh5*)‐Cre^ERT2^ × Rosa26‐tdTomato mice (supplementary material, Figure S12A). *Cdh5*‐Cre^ERT2^ × Rosa26‐tdTomato mice with or without four pulses of tamoxifen administration were used in the isografting model for inducible and irreversible labelling and tracing of ECs (CADH5^+^ cells) and their progeny in aortic graft arteriosclerosis (supplementary material, Figure S12A). Immunostaining confirmed the abundant accumulation of tdTomato^+^/SMαA^+^ cells in the neointimal lesions (supplementary material, Figure S12B), indicating that the donor ECs underwent an EndoMT process and that this process (or ECs undergoing EndoMT) contributed to isograft‐induced neointima formation. Importantly, no tdTomato/RFP signals were observed in the grafted aortas in the absence of tamoxifen, demonstrating no ‘leak’ of the *Cdh5*‐Cre^ERT2^ × Rosa26‐tdTomato system (supplementary material, Figure S12C).

**Figure 5:**
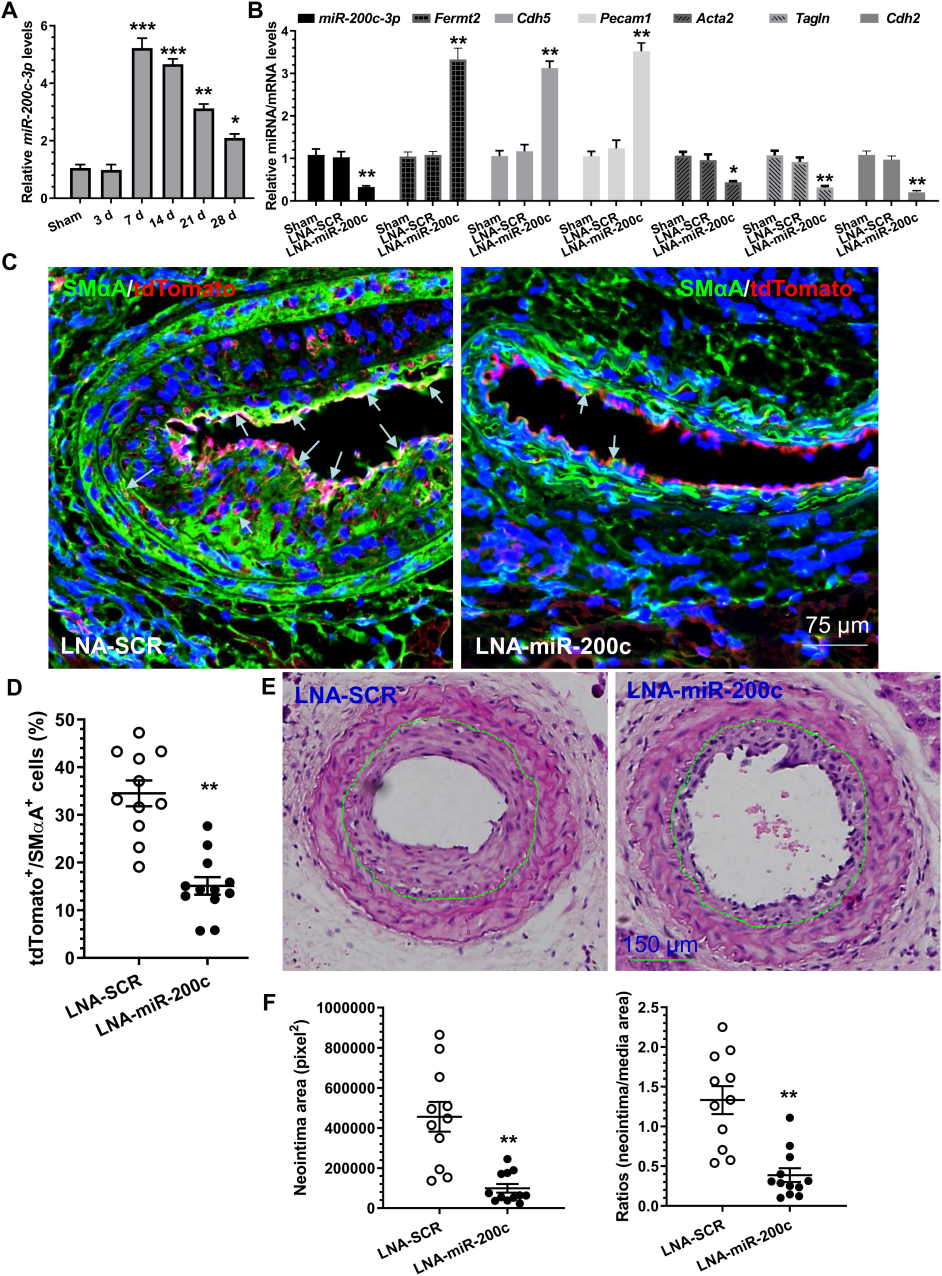
miR‐200c‐3p inhibition in grafted aortas reduces EndoMT and prevents neointima formation in the grafts. (A) Increased expression of miR‐200c‐3p in grafted aortas. Total RNAs were harvested from non‐implanted (used as sham surgery) and post‐implanted aortas at the indicated times and subjected to RT‐qPCR analyses. The data presented here are mean ± SEM of five independent experiments (aortas from 3–5 mice were pooled for each experiment, *n* = 5 experiments). *p < 0.05, **p < 0.01, ***p < 0.001 (versus sham; one‐way ANOVA with a Tukey's *post hoc* test). (B) miR‐200c‐3p inhibition prevents EndoMT in the grafted aorta. Two weeks after grafting, the grafted aortas from 6–8 mice were harvested, pooled, and prepared for cell sorting. Total RNAs were extracted from tdTomato^+^ cells isolated from the grafted aortas treated with medium only (Sham), a scrambled negative control (LNA‐SCR), or LNA‐miR‐200c‐3p (LNA‐miR‐200c) inhibitor, respectively, and subjected to RT‐qPCR analysis. The data presented here are mean ± SEM of five independent experiments (*n* = 5). *p < 0.05 (one‐way ANOVA with a Tukey's *post hoc* test). (C, D) Decreased numbers of cells underwent EndoMT in the grafted aortas treated with LNA‐miR‐200c. Four weeks after grafting, grafted aortas were harvested and subjected to immunostaining. The data presented here are the representative images (C) and the quantitative results of tdTomato^+^/SMA^+^ (cells underwent EndoMT) (D) from 11 (LNA‐SCR) and 12 (LNA‐miR‐200c) mice, respectively. Note:  arrows in C indicate cells that underwent EndoMT. (E, F) miR‐200c‐3p local inhibition decreases neointimal hyperplasia in vascular grafts. Four weeks after grafting, grafted aortas were harvested and prepared for H&E staining analyses. Representative images (E) and morphological characteristics (F) including neointimal area and neointimal/media (N/M) ratio of the implanted aortas from 11 (LNA‐SCR) and 12 (LNA‐miR‐200c) mice, respectively, are presented here. **p < 0.01 (versus LNA‐SCR, Student's *t*‐test).

After confirming the contribution of EndoMT to isograft‐induced neointima formation, we decided to examine the expression profile of miR‐200c‐3p in graft restenosis. We found that miR‐200c‐3p expression peaked at day 7 post‐grafting and gradually decreased thereafter (Figure 5A), indicating the involvement of miR‐200c‐3p in isograft‐induced neointima formation. To confirm such involvement, locked nucleic acid (LNA)‐modified oligonucleotides (LNA‐SCR, a scrambled negative control, or LNA‐miR‐200c, for miR‐200c‐3p specific inhibition) were directly infused into the lumen of the aortas immediately after harvesting to induce local endothelium transfection. Two weeks after grafting, tdTomato^+^ cells were isolated from the grafted aortas and subjected to RT‐qPCR analysis. The data shown in Figure 5B confirmed that miR‐200c‐3p expression was successfully inhibited in donor ECs and their progeny. Consistent with this, we observed increased expression of *Fermt2* along with EC markers (*Pecam1* and *Cdh5*), but decreased expression of mesenchymal/SMC genes (*Acta2*, *Tagln*, and *Cdh2*) in donor ECs of the grafted aorta transfected with LNA‐miR‐200c (Figure 5B), demonstrating a role for miR‐200c‐3p in EndoMT in the context of aortic graft remodelling. This was further supported by double immunofluorescence staining of the grafted aortas using antibodies against RFP/tdTomato and SMαA (Figure 5C,D). Similar to our *in vitro* observation, we observed that SRF protein was mainly accumulated within cell nuclei in the aortas treated with LNA‐SCR, but their main cellular location was cytoplasm in the aortas infected with LNA‐miR‐200c (supplementary material, Figure S13). Importantly, we observed an approximately 70% decrease in neointima formation in grafted aortas treated with LNA‐miR‐200c, compared with the aortas treated with LNA‐SCR (Figure 5E,F).

### Expression of the miR‐200c‐3p/FERMT2 regulatory axis in human arteries

**Figure 6:**
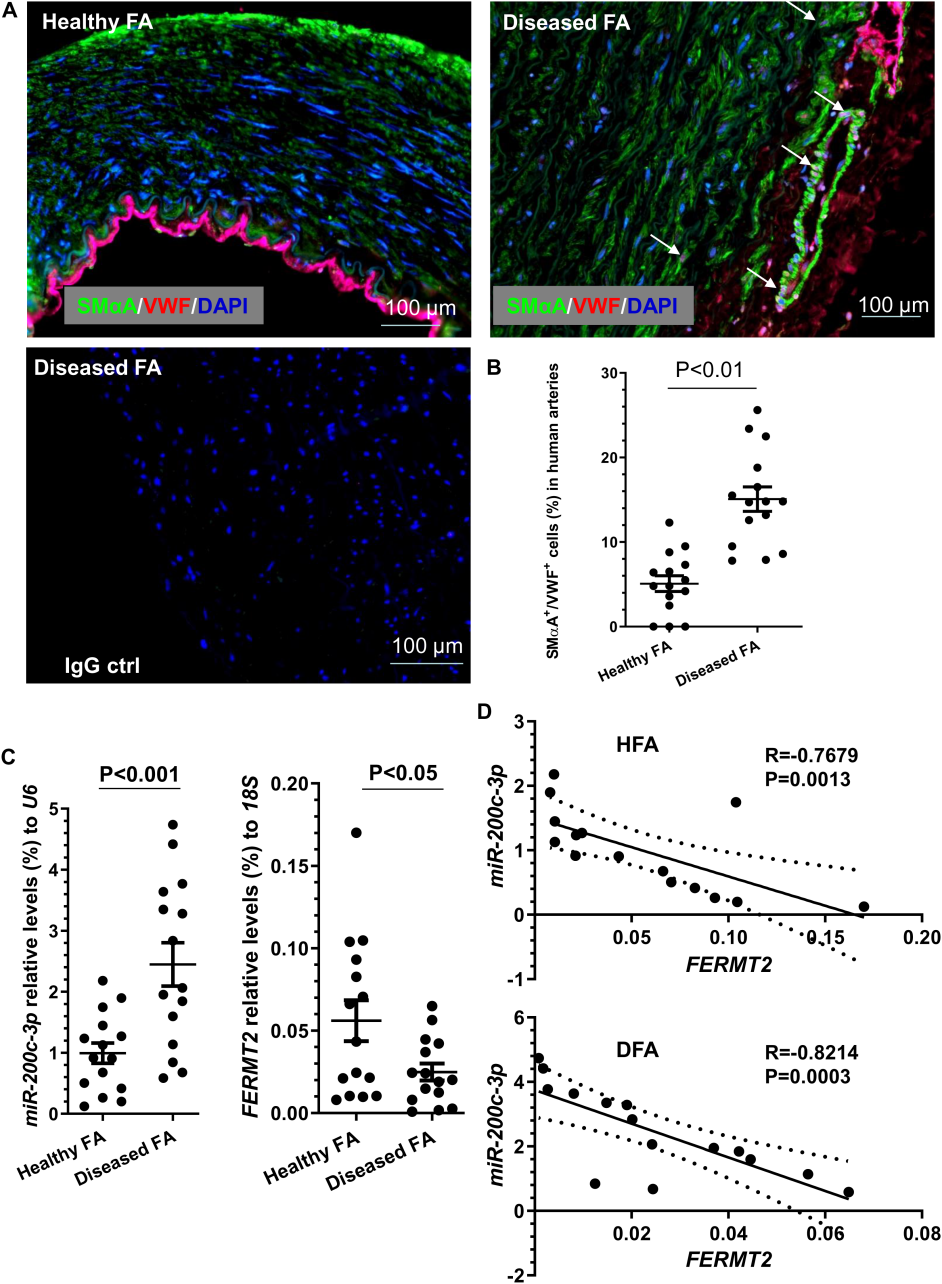
Detection of EndoMT and miR‐200c‐3p/FERM2 regulatory axis in human arteries. Human femoral arterial specimens from patients with (diseased FA, DFA) or without (healthy FA, HFA) peripheral arterial diseases who underwent leg amputation were collected and subjected to immunostaining assays (A, B) and RT‐qPCR analyses (C, D), respectively. (A, B) Higher incidence of EndoMT was detected in DFA. The data presented here are the representative images (A) and the quantitative results of VWF^+^/SMαA^+^ (suggestive of EndoMT) (B) from 15 patients (*n* = 15 patients for both groups). Student's *t‐*test. Note:  the arrows in A indicate cells undergoing EndoMT. (C) miR‐200c‐3p and *FERMT2* gene expression in human arteries. A Mann–Whitney *U*‐test was applied for statistical analysis. (D) Spearman's rank correlation coefficient analyses of the expression levels of miR‐200c‐3p and *FERMT2* in human femoral arterial specimens. *N* = 15 from each group.

We have so far demonstrated that the miR‐200c‐3p/FERM2 regulatory axis plays a critical role in EndoMT and aortic graft restenosis. To further validate these findings in a clinical setting, we first conducted double immunofluorescence assays in human femoral arterial specimens collected in our recent study [35]. Immunostaining showed a higher number of VWF^+^/SMαA^+^ cells, suggestive of cells undergoing EndoMT, in the diseased arteries, compared with the healthy arteries (Figure 6A,B and supplementary material Figure S14). Similarly, the cDNAs generated in the same study [35] were used for examining the expression levels of miR‐200c‐3p and *FERMT2* in these femoral arterial specimens by RT‐qPCR analysis. Compared with healthy arteries, an increased expression level of miR‐200c‐3p, but a decreased expression level of the *FERMT2* gene, was observed in the diseased arteries (Figure 6C). Additionally, we found a significant inverse relationship between miR‐200c‐3p and *FERMT2* gene expression levels in both healthy and diseased femoral arterial specimens (Figure 6D).

## Discussion

The major cause for vascular graft loss/failure after CABG is accelerated coronary allograft arteriosclerosis, characterized by neointimal hyperplasia consisting mainly of SMC‐/myofibroblast‐like cells. However, the cellular origins of these neointimal cells are still undergoing extensive debate [39, 40]. EndoMT has recently been suggested as an important driver of neointima formation in a murine transplant arteriopathy model and in rejecting human transplant lesions [6]. In agreement with this report, using an inducible genetic lineage tracing (Cdh5‐Cre^ERT2^ × Rosa26‐tdTomato mice) system and aortic isograft model, we provide genetic lineage tracing evidence that ECs undergoing EndoMT are critical contributors of neointimal cells in mouse isograft transplantation. Our immunostaining data also clearly provide evidence for the existence and involvement of EndoMT in human arteriosclerosis. Moreover, we have advanced our knowledge about the underlying molecular mechanisms of EndoMT by reporting a regulatory role for *miR‐200c‐3p* in promoting EndoMT *in vitro* and *in vivo*. Data generated from our mechanistic studies confirmed *FERMT2* as a functional target gene of miR‐200c‐3p in the context of EndoMT. Furthermore, we also provide clear evidence to support the notion that miR‐200c‐3p suppresses *FERMT2* gene expression in the grafted aortas, triggers the EndoMT process, and promotes aortic graft restenosis. Hence, the use of a miR‐200c‐3p inhibitor to dampen EndoMT processes in vascular grafts may be a reasonable interventional approach for vascular graft loss/failure after CABG (supplementary material, Figure S15).

An inhibitory role for the whole miR‐200 family in EMT and its inverse processes have been well established [28]. Recently, two miR‐200 family members (miR‐200a [41] and miR‐200b [42]) have been reported to play a similar inhibitory role in EndoMT, while the functional involvement of another three members (miR‐141, miR‐200c, and miR‐429) in EndoMT remains to be seen. To our surprise, in this study we have documented a promotive effect of miR‐200c‐3p on EndoMT and aortic graft remodelling. Through miR‐200c‐3p gain/loss‐of function analyses, we first demonstrated that miR‐200c‐3p promotes EndoMT, as evidenced by decreased expression of EC genes, increased expression of SMC‐like or mesenchymal genes, as well as acquired SMC‐like or mesenchymal cell morphology and function, and a simultaneous loss of EC functional characteristics (Figure 1 and supplementary material, Figures S3–S6). Importantly, by using a vascular graft‐induced neointima formation model and through local incubation of the endothelium of the grafts with LNA‐miR‐200c, we further demonstrated that miR‐200c‐3p inhibition reduces EndoMT and inhibits neointimal hyperplasia in the grafted arteries, showing that miR‐200c‐3p is a potential therapeutic agent in vascular grafting‐induced restenosis. Therefore, findings from this study and previous studies [30, 43] indicate that miR‐200c‐3p exerts an antagonist role in EMT (inhibiting) and EndoMT (promoting), and that miR‐200c‐3p can play divergent roles in different biological processes. Consequently, caution should be taken when considering the clinical applications for treating distinct human diseases through modulation of miR‐200c‐3p signalling.

A new finding from this study reveals that *FERMT2* is the functional and authentic target gene of miR‐200c‐3p in the context of EndoMT and aortic graft remodelling. Although a handful of miR‐200c‐3p target genes have been reported in various cellular contexts and diseases [29, 30], we have now provided several lines of evidence to support the notion that *FERMT2* is a novel target gene of miR‐200c‐3p during EndoMT, and have demonstrated that miR‐200c‐3p promotes EndoMT by targeting *FERMT2*. Our finding aligns perfectly with the functional implications of FERM2 in EMT and other cellular functions. Global deletion of the *FERMT2* gene causes embryonic lethality [44] and severe abnormalities of heart development [45, 46], and genetic deletion of *FERMT2* at late gestation or in adult cardiac myocytes results in heart failure and premature death because of enlargement of the heart and extensive fibrosis [47]. Later studies reported a role for FERM2 in epithelial cell phenotype modification [48], pathological and developmental angiogenesis [49], renal tubular cell plasticity [50] and renal fibrosis [51], and platelet responses and haemostasis [52]. It has also been reported that FERM2 promotes cancer angiogenesis and tumour progression [53], or breast cancer metastasis [54] by modulating EMT, and serves as a mechano‐responsive protein to link mechano‐environmental signalling to proline metabolism, promoting tumour growth [55]. A critical role for FERM2 in the control of adipogenesis, lipid metabolism, and bone homeostasis [56], chondrocyte differentiation program and chondrogenesis [57], and myogenic [58] as well as mesenchymal stem cell differentiation [59] has also been reported in recent studies.

Another novel finding in this study is the role of FERM2 in regulating SMC and EC gene expression through a transcriptional repression and post‐transcriptional activation mechanism, respectively. Specifically, we demonstrated that FERM2 transcriptionally represses SMC‐specific gene expression by interacting with SRF and modulating its cellular location during EndoMT. Increasing evidence has suggested that FERM2 regulates gene expression through its direct interaction with and by stabilizing one or more partner proteins, thus controlling their associated signalling pathways. It has been reported that FERM2 regulates cell adhesion and spreading by recruiting and interacting with paxillin [60], Arp2/3 [61], myosin light‐chain kinase [59], and integrin‐linked kinase [62], respectively. FERM2 has been shown to directly interact with and stabilize DNMT1, and to increase the occupancy of DNMT1 at the E‐cadherin (*CDH1*) promoter, thereby suppressing E‐cadherin expression [63]. It also interacts with vascular endothelial cadherin‐based complexes to support vascular barrier integrity [64]. FERM2 regulates integrin outside‐in signalling by direct binding with actin [65], talin/paxillin [66], α‐actinin‐2 [67], and/or integrin β1 [67]/β3 [68]. Interestingly, FERM2 forms a transcriptional complex with β‐catenin and TCF4 to enhance Wnt signalling [69], and physically interacts with both TGFβ type I receptor and Smad3 to activate TGF‐β/Smad signalling [51], respectively. Moreover, the underlying mechanism of controlling EC endocytosis and recycling of CD39 and CD73 during haemostasis was attributed to the direct interaction of FERM2 with clathrin heavy chain [52]. In this study, we provided clear evidence for the first time to show that FERM2 could directly interact with SRF and retain it within the cytoplasm in normal ECs. However, such interaction is disrupted when these ECs undergo EndoMT or when FERM2 protein is removed/reduced by miR‐200c‐3p expression. Consequently, SRF is released from the FERM2/SRF complex within the cytoplasm, and translocates into the nuclei, thereby triggering the SMC gene transcription program [70] and promoting EndoMT.

With regard to EC gene regulation by FERM2 during EndoMT, several convincing lines of evidence have been described and presented in this study to support the mechanistic findings that FERM2 controls EC gene expression through a post‐transcriptional mechanism. We documented a positive relationship between FERM2 expression levels and EC gene expression and presented strong evidence to show that FERM2 controls both *VWF* and *CDH5* mRNA stability. We further confirmed an *in situ* interaction between FERM2 and YBOX1 proteins, and demonstrated direct binding and enrichment of *VWF* and *CDH5* mRNAs to YBOX1. YBOX1 is a DNA/RNA‐binding protein controlling gene expression by regulating mRNA stabilization and splicing [71, 72]. Apart from its apparent function in gene regulation, studies reported a regulatory role of YBOX1 in EMT [73, 74], mesenchymal–endothelial transition [75], and EC function [76]. Interestingly, the secreted form of YBOX1 has also been implicated in controlling cell proliferation and migration [77]. We have now documented a novel role for YBOX1 in the regulation of EC gene expression and/or EndoMT by stabilizing EC gene transcripts. It has been suggested that YBOX1 regulates the stability of its target mRNA by recruiting ELAVL1 [72] or another RNA binding protein, nucleolin [71]. Therefore, further studies are warranted to investigate whether ELAVL1, nucleolin, or other co‐regulators are required for the preventive effect of the FERM2/YBOX1 complex on *VWF/CDH5* mRNA degradation during EndoMT.

Apart from the validated target gene, FERM2, an additional 40 proteins were found to be down‐regulated by miR‐200c‐3p overexpression during EndoMT in our proteomics analyses (Figure 2A and supplementary material, Figure S7A). Among them, 23 genes were predicted to contain one or more miR‐200c‐3p binding sites, and thus represent likely candidates which may potentially function as target genes for miR‐200c‐3p. As expected, some of them (e.g. *ZEB1*, *KLF9*, *SUZ12*) have been reported as target genes of miR‐200c‐3p in EMT and other biological processes, as summarized in two reviews [29, 30]. However, the functional implications of these genes in EndoMT and vascular grafting‐induced remodelling remain to be seen.

Taken together, we have demonstrated that miR‐200c‐3p plays an important role in EndoMT and aortic isograft‐induced restenosis and have identified FERM2 as the functional downstream target of miR‐200c‐3p in the context of EndoMT and vascular graft remodelling. These findings may provide novel insights into the pathogenesis of neointimal hyperplasia in the vascular grafts and have further potential therapeutic implications for vascular diseases (supplementary material, Figure S15). Since EndoMT plays a key role in the pathogenesis of various diseases, modulating the miR‐200c‐3p/FERM2 regulatory axis in the context of EndoMT may represent a broadly effective therapy strategy against a host of other fibrotic disorders including pulmonary, intestinal, cardiac, and kidney fibrosis.

## Author contributions statement

QX was responsible for conceptualization; DC, CZ and MY for methodology; and DC, CZ, JC, MY, TAA, WA, SH, JL and XW for investigation. QX wrote the original draft, and QX and EMM reviewed and edited the paper. QX was responsible for project administration. QX and QW acquired funding and were responsible for resources. YZ, QW and QX supervised the study.

## Supporting information


**Supplementary materials and methods**
**Figure S1.** EndoMT
**Figure S2.** Heatmap showing the miRNA expression profiles during EndoMT
**Figure S3.** miR‐200c‐3p inhibition prevents EndoMT
**Figure S4.** miR‐200c‐3p overexpression promotes EndoMT
**Figure S5.** Functional impacts of miR‐200c‐3p overexpression in EndoMT
**Figure S6.** Functional effects of miR‐200c‐3p modulation on EndoMT in HAoECs
**Figure S7.** Proteins and signalling pathways modulated by miR‐200c‐3p during EndoMT
**Figure S8.** *FERMT2* gene inhibition abolishes the promotive effect of miR‐200c‐3p knockdown on EndoMT
**Figure S9.** FERM2 (or FERMT2) protein–protein interaction (PPI) network retrieved from multiple PPI databases (e.g. BioGrid, CORUM, IntAct, MINT, and/or STRING)
**Figure S10.** FERM2 knockdown or miR‐200c overexpression causes SRF nuclear accumulation during EndoMT
**Figure S11.** FERM2 knockdown reduces FERM2 and YBX1 co‐localization
**Figure S12**. EndoMT contributes to neointima formation in aortic grafts
**Figure S13.** SRF cellular locations in the grafted aortas treated with LNA‐SCR or LNA‐miR‐200c
**Figure S14.** Individual images showing VWF^+^/SMαA^+^ (suggestive of EndoMT) cells in the two diseased human femoral arteries (A, B)
**Figure S15.** Schematic illustration showing the model of action for miR‐200c‐3p in EndoMT and neointimal SMC hyperplasia in vascular grafts
**Table S1.** Primer sets used in the present study (mentioned in the supplementary material, Supplementary materials and methods)

## Data Availability

The data that support the findings of this study are available on reasonable request.
