# Supplementary material for: miRNA‐200c‐3p promotes endothelial to mesenchymal transition and neointimal hyperplasia in artery bypass grafts
Source: J Pathol. 2020 Nov 28;253(2):209–24. doi: 10.1002/path.5574 (PMC7839516; doi:10.1002/path.5574)
Supplement: Supplementary file 1 — Supplementary materials and methods Figure S1. EndoMT Figure S2. Heatmap showing the miRNA expression profiles during EndoMT Figure S3. miR‐200c‐3p inhibition prevents EndoMT Figure S4. miR‐200c‐3p overexpression promotes EndoMT Figure S5. Functional impacts of miR‐200c‐3p overexpression in EndoMT Figure S6. Functional effects of miR‐200c‐3p modulation on EndoMT in HAoECs Figure S7. Proteins and signalling pathways modulated by miR‐200c‐3p during EndoMT Figure S8. FERMT2 gene inhibition abolishes the promotive effect of miR‐200c‐3p knockdown on EndoMT Figure S9. FERM2 (or FERMT2) protein–protein interaction (PPI) network retrieved from multiple PPI databases (e.g. BioGrid, CORUM, IntAct, MINT, and/or STRING) Figure S10. FERM2 knockdown or miR‐200c overexpression causes SRF nuclear accumulation during EndoMT Figure S11. FERM2 knockdown reduces FERM2 and YBX1 co‐localization Figure S12. EndoMT contributes to neointima formation in aortic grafts Figure S13. SRF cellular locations in the grafted aortas treated with LNA‐SCR or LNA‐miR‐200c Figure S14. Individual images showing VWF+/SMαA+ (suggestive of EndoMT) cells in the two diseased human femoral arteries (A, B) Figure S15. Schematic illustration showing the model of action for miR‐200c‐3p in EndoMT and neointimal SMC hyperplasia in vascular grafts Table S1. Primer sets used in the present study (mentioned in the supplementary material, Supplementary materials and methods) [file PATH-253-209-s001.doc]

**MiRNA-200c-3p promotes endothelial to mesenchymal transition and neointimal hyperplasia in artery bypass grafts**

D Chen, C Zhang *et al. J Pathol* DOI: 10.1002/path.5574

**Contents**

Supplementary materials and methods

Supplementary Figures S1–S15

Supplementary Table S1

**Supplementary materials and methods**

Reference numbers refer to the main text list

**Antibodies**

An antibody to FERM2/FERMT2/Kindlin-2 (mouse IgG, clone 3A3, MAB2617, 1 µg/ml for PLA and IF, 0.5 µg/ml for WB) was purchased from Sigma/Merck (Haverhill, UK). Antibodies against SRF (rabbit IgG, ab53147, 1 µg/ml for PLA and IF, 5 µg per IP for RIP), YBOX1 (rabbit IgG, ab12148, 1 µg/ml for PLA, 5 µg per IP for RIP), SMαA (mouse IgG, ab7817, 1 µg/ml), TAGLN/SM22α (rabbit IgG, ab14106, 1 µg/ml), VE Cadherin/CADH5 (rabbit IgG, ab33168, 1 µg/ml), VWF (rabbit IgG, ab6994, 1:500), S100A4 (rabbit IgG, ab197896, 1:1000), SNAIL (goat IgG, ab53519, 0.3 µg/ml), TWIST (mouse IgG, ab50887, 1 µg/ml), and GAPDH (mouse IgG, ab8245, 1:1000) were purchased from Abcam (Cambridge, UK). Antibody against RFP/tdTomato (rabbit IgG, 600-401-379, 1:1000) was from Rockland Immunochemicals Inc (Limerick, PA, USA). All secondary antibodies were from Thermo Fisher Scientific (Loughborough, UK). Other materials used in this study were purchased from Sigma/Merck (Haverhill, UK) unless otherwise specified.

**Cell culture and induction of EndoMT**

HUVECs from pooled donors (C2519A; Lonza, Basel, Switzerland) and human aortic endothelial cells (HAoECs, CC-2535; Lonza) were cultured in basal media (EGM-2 media that contained 2% fetal bovine serum, FBS; Lonza) and growth factor bullet kit (Lonza), or in M199 medium (Merck) that contained 20% FBS (Merck) and was supplemented with ECGS (endothelial cell growth supplement from bovine neural tissue, E2759, 5 µg/ml; Merck), beta-ECGF (β-endothelial cell growth factor human, E1388, 2.5 ng/ml; Merck), 2.5 µg/ml thymidine (Merck), 10 units/ml heparin (Merck), l-glutamine (Thermo Fisher Scientific), and penicillin/streptomycin (Thermo Fisher Scientific). A minimum of three different batches of HUVECs and HAoECs between passages 5 and 10 were used in this study. To induce EndoMT, six-well plates were pre-coated with 50 ng/ml fibronectin (F2006; Merck) for 1 h. HUVECs or HAoECs were initially seeded at 5 × 10^4^ cells per well overnight. EndoMT was initiated by culturing the cells in M199 medium supplemented with 20% FBS, 1% penicillin–streptomycin stock solution, 2 mm l-glutamine, 5 ng/ml TGFβ1 (Biolegend, London, UK), and 5 ng/ml TNFα (Biolegend) for up to 8 days. The medium was refreshed every 2 days.

**MiR-200c-3p inhibitor transfection**

Either control-scramble miRNA inhibitor or miR-200c-3p inhibitors (30 nm, final concentration) were transfected into HUVECs or HAoECs using TransIT-X2 Transfection Reagent (Geneflow Limited, Lichfield, UK) according to the manufacturer’s instructions. With Cy3™ Dye-Labeled Pre-miR Negative Control #1 (AM17120; Thermo Fisher Scientific), we detected over 70% of transfection efficiency for miRNA transfection in HUVECs. Both scramble and miR-200c-3p miRNA inhibitors were purchased from Thermo Fisher Scientific.

**Human *FERMT2* 3'-UTR clone and miR-200c-3p binding sites mutation.** The 3'-flanking untranslated region of the human *FERMT2* gene was amplified by PCR with primers shown in supplementary material, Table S1, and cloned into the Sac I and Mlu I sites of the vector pmiR-reporter-basic (Thermo Fisher Scientific), designated as pmiR-FERMT2-WT. MiR-200c-3p binding site 1/2 mutations alone or combinations were introduced into pmiR-FERMT2-WT by using a QuikChange™ site-directed mutagenesis kit (Agilent Technologies, Cheadle, UK) according to the manufacturer’s instructions. These were designated as pmiR-FERMT2-BS1^mut^, pmiR-FERMT2-BS2^mut^, and pmiR-FERMT2-BS1/2^mut^, respectively. All vectors were verified by DNA sequencing.

**Human SMC gene reporter plasmids and related SRF binding site mutants.** Human SMαA (*ACTA2*) and SM22α (*TAGLN*) gene promoter DNA were amplified by PCR from human genomic DNA with the primer set as shown in supplementary material, Table S1 and cloned into Xho I/Hind III sites of the pGL3-Enhancer expression vector (Promega, Southampton, UK), designated as pGL3-*ACTA2* and pGL3-*TAGL*N, respectively. SRF binding site mutation alone or combinations were introduced into pGL3-*ACTA2* or pGL3-*TAGLN* by using a QuikChange™ site-directed mutagenesis kit (Agilent Technologies) according to the manufacturer’s instructions. These were designated as pGL3-*ACTA2*-SRF^mut^ and pGL3-*TAGLN*-SRF^mut^, respectively. All vectors were verified by DNA sequencing.

**Transient transfection and luciferase activity assays**

Luciferase assays were conducted as described in our previous studies [31,35,78–82]. In brief, for gene promoter activity assays, HUVECs were co-transfected with individual gene promoters (pGL3-*ACTA2*, pGL3-*TAGLN*, pGL3-*ACTA2*-SRFmut, pGL3-*TAGLN*-SRF^mut^, pGL3-*VWF*, or pGL3-*CDH5*; the last two gene reporters [31] were generated in our previous study) (0.15 μg/2.5 × 10^4^ cells) and p*Renilla* (15 ng/2.5 × 10^4^ cells) using TransIT-X2 Transfection Reagent (Geneflow Limited), according to the manufacturer’s instructions. Transfected cells were subjected to various treatments as indicated in the respective figure legend. A Dual-Luciferase Reporter Assay System was used for detecting luciferase and *Renilla* activities according to the protocol provided in the system. Relative luciferase unit (RLU) was defined as the ratio of luciferase versus *Renilla* activity with that of the control (set as 1.0).

For *FERMT2* 3'-UTR reporter activity assays, HUVECs infected with control or *miR-200c-3p* overexpression lentivirus were transfected with individual reporter genes (pmiR-*FERMT2*-WT, pmiR-*FERMT2*-BS1mut, pmiR-*FERMT2*-BS2mut, and pmiR-*FERM*T2-BS1/2mut, 0.15 μg/2.5 × 10^4^ cells) using TransIT-X2 Transfection Reagent (Geneflow Limited), according to the manufacturer’s instructions. pmiR-Luc-β-gal (0.20 μg/2.5 × 10^4^ cells) was included in all transfection assays as internal controls. Luciferase and β-galactosidase activities were detected 48 h after transfection using a standard protocol. Relative luciferase unit (RLU) was defined as the ratio of luciferase versus β-galactosidase activity with that of the control (set as 1.0).

***FERMT2* shRNA lentivirus**

Lentiviral particles were produced using MISSION shRNA *FERMT2* plasmids DNA (SHCLNG-NM_006832, Merck) according to the protocol provided. The shRNA Non-Targeting control vector (SHC002) was used as a negative control. *FERMT2* shRNA lentiviral infection was performed as described in our previous studies [83–85]. In brief, HUVECs were plated 24 h prior to infection in six-well plates at 37 °C. One transducing unit per cell (or 2–3 × 10^5^ per well) of shRNA or control virus was added with 10 μg/ml hexadimethrine bromide (H9268; Merck). Viral constructs were incubated for 24 h with the cells before the media were replaced with complete media.

**Generation of *miR-200c-3p* overexpression pseudo-viral particles**

The genomic DNA of *HSA-miR-200c-3p* (MI0000650) including the stem-loop sequence and its 5'-/3'-flanking sequence (~860 bp) was amplified by PCR with the primers shown in supplementary material, Table S1, and cloned into the Hpa I and Xho I sites of pLL3.7-*GFP* (Plasmid 11795; Addgene, Watertown, MA, USA), designated as Lenti-miR-200c. Similarly, a scrambled DNA sequence with a similar length was generated using Random DNA Sequence Generator (https://faculty.ucr.edu/~mmaduro/random.htm) and synthesized by GenScript (Nanjing, Jiangsu, PR China). The resultant DNA fragment flanking with the sequences recognized by Hpa I and Xho I was sub-cloned into pLL3.7-*GFP* vector, designated as Lenti-SCR. 293T cells were transfected with the SCR or *miR-200c-3p* plasmid and the packaging plasmids pMDLg/pRRE (Addgene, 12251), pRSV-Rev (Addgene, 12253), and pMD2.G (Addgene, 12259) using Turbofect transfection reagent (Thermo Fisher Scientific), according to the manufacturer’s instructions. The supernatant containing the lentivirus was harvested 48 h later, filtered, aliquoted, and stored at –80 °C. For lentiviral infection, HUVECs were plated 24 h prior to infection in T25 flasks at 37 °C. One millilitre of respective lentiviral particles was added with 10 μg/ml hexadimethrine bromide (H9268; Merck). Viral constructs were incubated for 24 h with the cells before the media were replaced with complete media.

**Cellular functional assays**

**Cell proliferation (CCK-8) assays.** Cell Counting Kit-8 (CCK-8) kit (Sigma/Merck, 96992-500TESTS-F) was used to measure cell proliferation after the cells received various treatments as indicated in the respective figure (figure legend), according to the manufacturer’s instructions.

**Transwell migration assays.** Cells with various treatments as indicated within the respective figure (figure legend) were cultured in FBS-free M199 medium overnight and harvested by trypsinization. An aliquot (250 000 cells per 200 µl) of the cells in serum-free M199 was dispensed into the Transwell inserts (8 µm pore size; Greiner Bio-One Ltd, Stonehouse, UK; Item number: 662638) pre-coated with 0.5% gelatin (Merck, G1393), and M199 (Merck) with 20% FBS was placed in the lower chamber. The Transwell plates were incubated at 37 °C in a 5% CO_2_ incubator for 12–18 h. Non-migrated cells in the top insert were carefully removed using a cotton swab, and the migrated cells in the bottom side were stained with Crystal Violet dye. Images were captured at five fixed locations (right, bottom, left, up, and centre), and migrated cells were counted by two experienced investigators blinded to the treatments.

**Tube formation**

Pre-chilled 96-well plates were coated with 50 µl of Matrigel^TM^ Basement Membrane Matrix (BD Biosciences, Bedford, MA, USA) per well and incubated for 30 min at 37 °C. 1 × 10^5^ cells with various treatments were counted and plated into each well. These cells were allowed to settle for another 30 min in the incubator at 37 °C. Thereafter, 400 µl of EC culture medium as described in the previous section was added to the settled cells and the formation of tubes was observed 2–24 h later using an EVOS® FL Auto Imaging System (Thermo Fisher Scientific). ImageJ software with an Angiogenesis Analyzer plugin [National Institutes of Health (NIH), ‎Bethesda, MD‎, USA] was used to analyse the master branch points, master branches, and total tube/branch length within each image.

**Western blotting**

Cells were harvested and lysed in lysis buffer (50 mm Tris-Cl, pH 7.5; 150 mm NaCl; 1 mm EDTA, pH 8.0) supplemented with protease inhibitors and 0.5% Triton, and sonicated to obtain whole cell lysate. Forty micrograms of protein was separated by SDS-PAGE using a 4–20% Tris-Glycine gel (Thermo Fisher Scientific) and subjected to standard western blotting analysis. The blots were subjected to densitometric analysis using ImageJ software (NIH). Relative protein expression level was defined as the ratio of target protein expression level to GAPDH expression level.

**Proteomics studies and data analysis**

Proteomics studies and data analysis were conducted as described in our previous study [79]. In brief, after 4 days of EndoMT, HUVECs infected with Lenti-SCR or Lenti-*miR-200c-3p* were directly lysed in a urea-based lysis buffer and proteins were digested using trypsin. Protein-derived peptides were analysed by an LTQ Orbitrap XL mass spectrometer (Thermo Fisher Scientific) coupled to a nanoAcquity LC (Waters). MS/MS data were converted to mgf files using Mascot Distiller (version 2.2; Matrix Science Ltd, London, UK) and searched against UniProt SwissProt using the Mascot search engine (version 2.2; Matrix Science Ltd). Significance of peptide identification was assessed by comparing results returned by searches against random and forward databases. Fold discovery rates at several cut-off values of Mascot scores and mass tolerances were used to calculate an empirical value of probability of random identification. Relative quantification of peptides across experimental conditions was achieved by comparing peak heights of extracted ion chromatograms (automated by Pescal; Matrix Science Ltd). The data were normalized to the sum of all intensities derived from a sample (columns). When comparing the effects of *miR-200c-3p* overexpression on protein regulation, peptide signals were divided by those of the untreated control samples (Lenti-SCR). The *P* values of differences across treatments were obtained by means of a *t*-test of log_2_ transformed fold-changes and these were adjusted for multiple testing through the Benjamini–Hochberg procedure. The fold-change was transformed using the log_2_ function, so that the data were centred around zero, while the Benjamini–Hochberg corrected *P* value was −log_10_ transformed for the volcano plot analysis.

**Reverse transcription–quantitative real-time-PCR (RT-qPCR)**

RT-qPCR was performed as described previously [31,80,81]. In brief, total RNAs including small RNAs were extracted from cells/tissues using TRI reagent (Merck) according to the manufacturer’s instructions and subjected to DNase I (Merck) digestion to remove potential DNA contamination. Reverse transcription was performed using an Improm-IITM RT kit (Promega) with RNase inhibitor (Promega), and Random primers (Promega) for mRNAs. The NCode™ VILO™ miRNA cDNA Synthesis Kit (Thermo Fisher Scientific) was used to synthesize poly(A) tails on all miRNAs, followed by cDNA synthesis from the tailed population in a single reaction as described in our previous studies [31,35,78–82]. The resultant cDNA was diluted to a working concentration of 5 ng/μl and stored at −20 ºC. Relative miRNA or mRNA expression levels were defined as the ratio of target miRNA/gene expression level to *U6/18S* expression level, respectively, with that of the control sample set as 1.0. Primers were designed using Primer Express software (Thermo Fisher Scientific); the sequence for each primer is listed in supplementary material, Table S1.

**miProfile™ Custom miRNA qPCR Arrays analysis**

Sixty-seven miRNAs that have been reported to be involved in (associated with) either EMT or EndoMT were manually curated from publications and used for preparing the miProfile™ Custom miRNA qPCR Arrays in a 96-well plate format (GeneCopoeia, Rockville, MD, USA). The All-in-One™ miRNA RT-qPCR reagent kits including the All-in-One™ miRNA RT-qPCR Detection Kits and the All-in-One™ miRNA First-Strand cDNA Synthesis Kits (GeneCopoeia, QP016) were used for detecting miRNA expression profiles, according to the manufacturer’s instructions.

**Chromatin immunoprecipitation (ChIP) assays**

The ChIP assays were performed as described in our previous studies [31,80,81,84,86–88]. Cells with the indicated treatments were treated with 1% (v/v) formaldehyde at room temperature for 10 min and then quenched with glycine at room temperature. The medium was removed, and cells were harvested and sonicated. The sheared samples were diluted into 1 ml of immunoprecipitation buffer containing 25 mm Tris–HCl (pH 7.2), 0.1% NP-40, 150 mm NaCl, 1 mm EDTA, and immunoprecipitation was conducted with 5 µg of antibody raised against SRF (rabbit IgG, ab245462; Abcam), together with single-strand salmon sperm DNA saturated with protein-G-Sepharose beads (Thermo Fisher Scientific). Normal rabbit IgG was used as a control. The immunoprecipitates were eluted from the beads using 100 μl of elution buffer (50 mm NaHCO_3_, 1% SDS). A total of 200 μl of proteinase K solution was added to a total elution volume of 300 μl and incubated at 60 °C overnight. Immunoprecipitated DNA was extracted, purified, and then used to amplify target DNA sequences by qPCR. Promoter DNA enrichment with specific antibody was calculated using percent input method with that of the IgG control set as 1.0. The relative level of promoter DNA enrichment was defined as the ratio of promoter DNA enrichments in the samples with treatment(s) (sh-*FERM2*) to the control samples (sh-NT), with that of the control sample set as 1.0. PCR amplification of the adjacent promoter regions was included as additional control for specific promoter DNA enrichment.

**RNA immunoprecipitation (RIP) assays**

The procedure for RIP assays was conducted as described in our previous studies [78,81]. In brief, cells with various treatments were treated with 1% (v/v) formaldehyde at room temperature for 10 min and then quenched with glycine at room temperature. Cells were harvested, lysed, and sonicated in lysis buffer containing RNase inhibitor. The sheared samples were diluted into 1 ml of immunoprecipitation buffer, and immunoprecipitations were conducted with 5 µg of YBOX1 antibody (Abcam, ab12148) or an equal amount of rabbit IgG, together with protein-G Dynabeads® saturated with single-strand salmon sperm DNA (Thermo Fisher Scientific). The immunoprecipitates were digested with DNAse and eluted from the beads using 100 μl of elution buffer, and immunoprecipitated RNA was extracted, purified, and then used to amplify target RNA sequences by RT-qPCR using specific primers (supplementary material, Table S1). mRNA enrichment with specific antibody was calculated using the percent input method with that of the IgG control set as 1.0. PCR amplification of the VWF/CDH5 intron region was included as additional control for specific RNA enrichment.

**Aortic grafting experiments**

Mouse aortic isograft transplantation was performed as described previously with slight modifications [33,34]. In brief, thoracic aortic segments (~1 cm) were harvested from donor male mice (*Cdh5*-CreERT2 × Rosa26-tdTomato mice pretreated with or without tamoxifen), washed, and suspended with saline solution containing heparin (100 U/ml). The anaesthesia of recipient C57BL/6J female mice (GemPharmatech, Jiangsu, China) was induced using 100% O_2_–4% isoflurane and maintained throughout the procedure by the administration of 100% O_2_–2% isoflurane. The abdominal wall was incised at the midline from xyphoid to pubis, and spread apart using a micro-retractor to expose the abdominal cavity. The intestines, rectum, and reproductive organs were retracted/moved aside to expose the infrarenal aorta. The isolated segment of abdominal aorta was cross-clamped using two vascular clamps approximately 5 mm apart, and the anastomotic sites were created by transecting the abdominal aorta between the clamps using sharp micro-scissors. After a small segment of abdominal aorta was resected from the anastomotic sites, the aortic segments from donor mice were then implanted between two cut ends of abdominal aorta of donor mice. It is worth mentioning that graft rejection in this sex-mismatched isograft mouse model is only triggered by minor histocompatibility antigens encoded by the Y chromosome (present in the male donor mice only), which could preserve donor-derived vascular cells for several weeks, thereby allowing us to trace the donor-derived endothelial cells in the grafted arteries.

The procedures for local miR-200c-3p inhibition in the grafted aortas were similar to that described in our previous study [35] with some modifications. In brief, immediately after harvest, 50–60 µl of DMEM containing vehicle (mock transfection, sham), control scrambled locked nucleic acid (LNA)-modified oligonucleotides (LNA-SCR), or LNA-miR-200c-3p per vessel was randomly injected into the arteries, followed by a 30-min incubation for local endothelium transfection. After that, aortic segments were transplanted into the carotid artery using end-to-end anastomosis as described above. LNA-miR-200c-3p and LNA-SCR were purchased from Thermo Fisher Scientific (EXIQON, Loughborough, UK) with their corresponding sequences: 5'-TCCATCATTACCCGGCAGTATT-3' and 5'-ACGTCTATACGCCCA-3'. Grafted arterial fragments were harvested for gene expression assays or cell sorting at indicated time points. Perfusion-fixed grafted arteries were collected 28 days after grafting for paraffin embedding, H&E staining, immunostaining assays, and neointima lesion characterization. The investigators harvesting the tissues and collecting the data were blinded to the *miR-200c-3p* treatment.

**Morphometric analysis and quantification of lesion formation**

The grafted arteries were harvested at 4 weeks post-grafting. The specimens were fixed in 4% formaldehyde for H&E staining. Sections (8 µm) were collected from the anastomotic site at 200-µm intervals and numbered. Three digitized sections with the same identification number from three segments/intervals (~0.2, 0.4, and 0.6 mm from the anastomotic site) of each animal (e.g. I-1/2 and III-1/2 represent the first and second section of the first and third segment/interval, respectively) were stained with H&E for morphometric analysis. The procedure used for lesion quantification was similar to that described in our previous studies [78,82,89–92]. In brief, EEL (external elastic membrane), IEL (internal elastic membrane), lumen, media, and neointimal areas were automatically measured on H&E-stained cross-sectional carotid artery segments using a computerized image analysis system (pixel^2^, Axiovision software; Carl Zeiss AG, Oberkochen, Germany) by two experienced investigators blinded to the treatments. Three sections were analysed per vessel sample and averaged.

**Immunofluorescence analysis**

For immunofluorescence staining of mouse aortic tissues or human femoral arteries, paraffin sections of mouse aortic tissues (generated in this study) or human femoral arteries (collected from the First Affiliated Hospital of Zhejiang University, China, and described in detail in our previous study [35]) were deparaffinized with xylene and rehydrated with ethanol, and then incubated with 10 mm sodium citrate at 100 °C for 10 min to retrieve antigens, followed by incubation with a blocking solution (10% normal matched serum) for 30 min. Thereafter, the sections were incubated with indicated primary antibodies (RFP/tdTomato, 1:1000 and SMαA for mouse tissues, or VWF and SMαA for human tissues; all were 1:500 dilutions) or respective IgG controls diluted in blocking buffer in a cold room (4 °C) overnight. The tissue sections were then washed and subsequently incubated with an appropriate fluorescence-conjugated secondary antibody (1:1000 dilution), followed by staining of nuclei with 4,6-diamidino-2-phenylindole (DAPI) (1 µg/ml). After mounting, the slides were examined using a laser scanning confocal microscope (Zeiss LSM 510 Mark 4) and Zen 2009 image software (Carl Zeiss AG). Human tissue collection was approved by the Research Ethics Committees of the First Affiliated Hospital of Zhejiang University (Institutional Review Board approval No 2013/150), and all experiments were conducted according to the principles expressed in the Declaration of Helsinki.

For cultured cells, cells with various treatments were fixed with 4% paraformaldehyde (PFA) and subjected to double immunostaining with antibody against FERM2 together with antibody against either SRF or YBOX1 (all were 1 µg/ml), or respective IgG controls. After labelling with an appropriate fluorescence-conjugated secondary antibody (1:1000 dilution), cells were incubated with DAPI (1 µg/ml) and examined using a confocal microscope. Images were taken using an attached camera and processed with Photoshop software (Adobe, San José, CA, USA).

**Aortic cell sorting**

The procedures for mouse aortic cell (tdTomato^+^ cell) preparation [93] and isolation [90,94]. were similar to the protocol described in our previous studies, with slight modifications. In brief, for each experiment, the implanted arteries were harvested from 6–8 mice and cut into pieces 0.5–1 mm in size. The pooled tissue blocks were incubated with 3 mg/ml type II collagenase in DMEM with a 1/5 (w/v) ratio of tissue (g) to enzyme solution (ml). After incubation for 30 min, the same volume of 1 mg/ml elastase solution was added to the solution containing the tissue and collagenase. The tissues were incubated for another 1–2 h until all the tissues were digested. After filtering with a cell strainer (70 µm), single cell digestion solution was centrifuged to remove the digestion solution. These single cells were incubated with antibody against tdTomato/RFP on ice overnight. After washing, cells were incubated with 100 µl of Anti-Rabbit IgG MicroBeads (130-048-602; Miltenyi Biotec, Woking, UK) on ice for 2 h. After that, tdTomato^+^ cells were sorted by the MACS Manual Separator (Miltenyi Biotec) using similar procedures to those described in our previous studies [90,94].

**Proximity ligation assays (PLAs)**

PLAs were conducted using a Duolink® In Situ Red Starter Kit Mouse/Rabbit as described in our previous study [78]. In brief, cells were fixed with 4% PFA and permeabilized with 0.1% Triton X-100 in PBS for 30 min at room temperature. After washing with PBS twice, cells were blocked with 10% donkey serum for 30 min at room temperature and incubated with antibodies against FERM2 (mouse, 1 µg/ml)/SRF (rabbit, 1 µg/ml), FERM2 (mouse, 1 µg/ml)/YBX1 (rabbit, 1 µg/ml), or mouse IgG/rabbit IgG, at 4 °C overnight. Cells were then washed and subsequently incubated with the Duolink® In Situ PLA® Probe Anti-Mouse PLUS Affinity purified Donkey anti-Mouse IgG (H+L)/Duolink® In Situ PLA® Probe Anti-Rabbit MINUS Affinity purified Donkey anti-Rabbit IgG (H+L) (1:5 in 10% donkey serum in PBS) in a pre-heated humidity chamber for 1 h at 37 °C. After washing with PBS twice, cells were incubated with Ligation-Ligase solution in a pre-heated humidity chamber for 30 min at 37 °C, followed by incubation with Amplification-Polymerase solution in a pre-heated humidity chamber for 100 min at 37 °C. After that, the cells were washed three time in PBS and stained with 4,6-diamidino-2-phenylindole (DAPI) (1 μg/ml) for 5 min at room temperature. After mounting, cells were examined using a laser scanning confocal microscope (Zeiss LSM 510 Mark 4) and Zen 2009 image software (Carl Zeiss AG).

**Supplementary Figures S1–S15**

**Figure S1. EndoMT.** HUVECs were incubated with 5 ng/ml TGFβ1 and 5 ng/ml TNFα for the indicated times to induce EndoMT. (A) Phase-contrast images were captured, and total RNAs and proteins were harvested at the indicated times, followed by (B–D) RT-qPCR and (E, F) western blotting analyses. The data presented here are representative images (A; left panels of E, F) or mean ± SEM (B–D; right panels of E, F) of three to five (*n* = 3–5) independent experiments. **p*< 0.05, ***p*< 0.01, ****p*< 0.001 (versus D0; one-way ANOVA with a Tukey’s *post hoc* test).


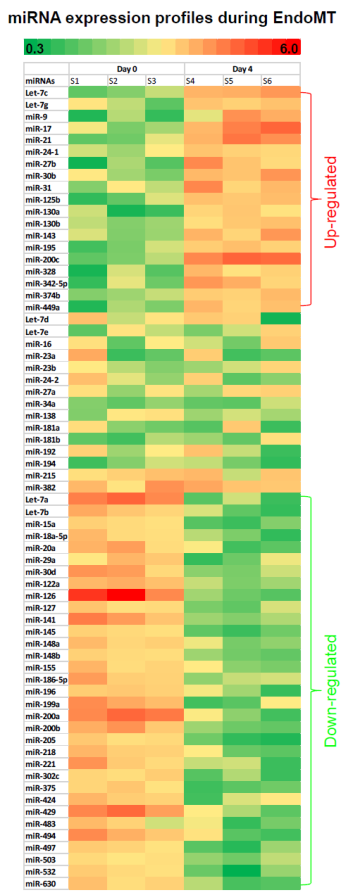
**Figure S2. Heatmap showing the miRNA expression profiles during EndoMT.** HUVECs were incubated with 5 ng/ml TGFβ1 and 5 ng/ml TNFα for 4 days to induce EndoMT. Normally cultured HUVECs (day 0) were used as a control. Total RNAs including small RNAs were extracted and subjected to the miProfile™ Custom miRNA RT-qPCR Arrays analysis. The data presented here were generated from three independent experiments (*n* = 3).

**Figure S3. miR-200c-3p inhibition prevents EndoMT.**

HUVECs were transfected with reagent control only (Ctrl), a scrambled negative control miRNA inhibitor (SCR inhibitor) or miR-200c-3p inhibitor (miR-200c inhibitor), respectively. Transfected cells were incubated with 5 ng/ml TGFβ1/TNFα for 4 days. After the phase-contrast photomicrographs in A were taken, cells were subjected to serum starvation overnight, followed by various assays as indicated. (B) CCK-8 analysis to detect cell proliferation in the absence (vehicle) or presence of 10 ng/ml PDGF-BB, respectively. (C) Transwell migration assay to examine the effect of miR-200c-3p inhibition on cell migration in the presence of 20% FBS. Note: in the Transwell migration experiments, only a few cells migrated through the insert without any chemoattractant. Representative photomicrographs (left) and quantitative results of the migrated cells (right) are presented here. (D, E) Matrigel tube formation assays. Harvested cells were seeded onto Matrigel-coated 96-well plates (1.5 × 10^4^ per well) for 12 h, and images were taken. (D) Representative photomicrographs and (E) quantitative data of master branch points, master branches, and total tube/branch length are presented. The data presented here were generated from five independent experiments (*n*= 5). ***p*< 0.01 (versus Ctrl/SCR), ^#^*p*<0.05 (PDGF-BB versus vehicle). One-way (C, E) or two-way (B) ANOVA with a Tukey’s *post hoc* test was used for statistical analysis.

**Figure S4. miR-200c-3p overexpression promotes EndoMT.** HUVECs were infected with medium control only (Ctrl), a scrambled negative control miRNA (Lenti-SCR) or miR-200c-3p (Lenti-miR-200c) lentiviral particles, respectively. Infected cells were incubated with 5 ng/ml TGFβ1/TNFα for 4 days to induce EndoMT. Total RNAs including small RNAs and proteins were collected and subjected to (A) RT-qPCR and (B) western blotting analysis, respectively. In panel B, representative images are shown on the left, and the quantitative results of blots on the right. The data presented here are mean ± SEM of five (*n* = 5) independent experiments. ***p*< 0.01, or ****p* < 0.001 (versus Ctrl/SCR; one-way ANOVA with a Tukey’s *post hoc* test).

**Figure S5. Functional impacts of miR-200c-3p overexpression in EndoMT.**

HUVECs received similar treatments to those described in Figure S4 and were subjected to similar assays detailed in Figure S3. (A) Phase-contrast photomicrographs. (B) CCK-8 assay. (C) Transwell migration. (D, E) Matrigel tube formation. The data presented here were generated from five independent experiments (*n* = 5). **p*< 0.05, ***p* < 0.01 (versus Ctrl/SCR), ^#^*p* < 0.05 (PDGF-BB versus vehicle). One-way (C, E) or two-way (B) ANOVA with a Tukey’s *post hoc* test was used for statistical analysis.

**Figure S6.** **Functional effects of miR-200c-3p modulation on EndoMT in HAoECs.**

(A) HAoECs were transfected with a scrambled negative control miRNA inhibitor (SCR inhibitor) or miR-200c-3p inhibitor (miR-200c inhibitor), or (B) infected with a scrambled negative control miRNA (Lenti-SCR) or miR-200c-3p (Lenti-miR-200c) lentiviral particles. (A) Transfected or (B) infected cells were incubated with 5 ng/ml TGFβ1/TNFα for 4 days to induce EndoMT. Total RNAs including small RNAs were collected and subjected to RT-qPCR analysis with the indicated primers. The data presented here were generated from five independent experiments (*n* = 5). ***p*< 0.01, ***p < 0.001 (versus SCR inhibitor or Lenti-SCR; Student’s *t*-test).

**Figure S7. Proteins and signalling pathways modulated by miR-200c-3p during EndoMT.** HUVECs infected with a scrambled negative control miRNA (Lenti-SCR) or miR-200c-3p overexpression (Lenti-miR-200c) lentivirus were incubated with 5 ng/ml TGFβ1/TNFα for 4 days to induce EndoMT. Total proteins were harvested and subjected to label-free quantitative proteomics analysis. (A) Heatmap showing all the proteins that were significantly regulated by miR-200c-3p overexpression during EndoMT. (B,C) PANTHER analysis of the significant down- and up-regulated proteins showing the fold-enrichments of (B) molecular functions and (C) biological processes modulated by miR-200c-3p overexpression during EndoMT.

**Figure S8. *FERMT2* gene inhibition abolishes the promotive effect of miR-200c-3p knockdown on EndoMT.**

HUVECs infected with a non-target (sh-NT) or *FERMT2* gene-specific shRNA (sh-FERMT2) lentivirus were transfected with a scrambled negative control miRNA inhibitor (SCR inhibitor) or miR-200c-3p inhibitor (miR-200c inhibitor) as indicated in the figures, followed by incubation with 5 ng/ml TGFβ1/TNFα for 4 days. Total RNAs were harvested and subjected to RT-qPCR analyses to examine the gene expression levels of miR-200c-3p and *FERMT2* (A), EC (B), and mesenchymal/SMC markers (C), respectively. The data presented here are mean ± SEM of five independent experiments (*n* = 5). **p*< 0.05, ***p*< 0.01 (versus SCR inhibitor/sh-NT), ^#^*p*< 0.05 (versus miR-200c inhibitor/sh-NT), one-way ANOVA with a Tukey’s *post hoc* test.

**Figure S9. FERM2 (or FERMT2) protein–protein interaction (PPI) network retrieved from multiple PPI databases (e.g. BioGrid, CORUM, IntAct, MINT, and/or STRING).**

SRF, serum response factor; YBX1, Y-box binding protein-1.

**Figure S10. FERM2 knockdown or miR-200c overexpression causes SRF nuclear accumulation during EndoMT.**

(A) HUVECs infected with a non-target (sh-NT) or FERM2 gene-specific shRNA (sh-FERM2) lentivirus, or (B) a scrambled negative control miRNA (Lenti-SCR) or miR-200c-3p (Lenti-miR-200c) lentiviral particles, were incubated with 5 ng/ml TGFβ1/TNFα for 4 days to induce EndoMT. Cells were fixed and subjected to immunostaining with the indicated antibodies. Representative images from five experiments (*n* = 5) are presented here.

**Figure S11. FERM2 knockdown reduces FERM2 and YBX1 co-localization.**

HUVECs infected with a non-target (sh-NT) or FERM2 gene-specific shRNA (sh-FERM2) lentivirus were incubated with 5 ng/ml TGFβ1/TNFα for 4 days to induce EndoMT. Cells were fixed and subjected to immunostaining with the indicated antibodies. Representative images from five experiments (*n* = 5) are presented here.

**Figure S12. EndoMT contributes to neointima formation in aortic grafts.**

(A) Schematic diagram illustrating animal procedures. (B, C) Immunofluorescence assays to detect cells that underwent EndoMT (tdTomato^+^/SMA^+^ cells) in the grafted aortas. Aortas isolated from male Cdh5-Cre^ERT2^ × Rosa26-tdTomato mice pretreated (B) with or (C) without tamoxifen were implanted into female C57/6J mice. Four weeks after grafting, grafted aortas were harvested and prepared for double immunostaining analyses with antibodies against smooth muscle α-actin (SMαA) and RFP/tdTomato. Representative images from five mice (*n* = 5) are presented here. Note: arrows indicate double-positive cells.

**Figure S13. SRF cellular locations in the grafted aortas treated with LNA-SCR or LNA-miR-200c.**

Four weeks after grafting, grafted aortas were harvested and subjected to immunostaining using an antibody against SRF. Representative images from five mice (*n* = 5) are presented here.

**Figure S14. Individual images showing VWF^+^/SMαA^+^ (suggestive of EndoMT) cells in the two diseased human femoral arteries (A, B).**

**Figure S15. Schematic illustration showing the model of action for miR-200c-3p in EndoMT and neointimal SMC hyperplasia in vascular grafts**.

MiR-200c-3p promotes EndoMT by targeting and inhibiting fermitin family member 2 (*FERM2*), which increases smooth muscle cell (SMC) gene expression by promoting serum response factor (SRF) nuclear translocation and increases EC mRNA decay by disrupting its interaction with Y-box binding protein 1 (YBOX1). Thus, miR-200c-3p inhibition prevents EndoMT and inhibits neointima formation in grafted arteries.

**Table S1. Primer sets used in the present study**

| **Gene** | **Forward (5'-3')** | **Reverse (5'-3')** | **Application** |
| --- | --- | --- | --- |
| *18S/18S* | AAACGGCTACCACATCCAAG | CCTCCAATGGATCCTCGTTA | RT-qPCR |
| *U6* | gatgacacgcaaattcgtg | miRNA universal reverse primer (Invitrogen, A11193-051) | RT-qPCR |
| *Pecam1* | CAAACAGAAACCCGTGGAGATG | ACCGTAATGGCTGTTGGCTTC | RT-qPCR |
| *Cdh5* | AAGAAACCGCTGATCGGCA | TCGGAAGAATTGGCCTCTGTC | RT-qPCR |
| *Acta2* | TCCTGACGCTGAAGTATCCGAT | GGCCACACGAAGCTCGTTATAG | RT-qPCR |
| *Tagln* | GATATGGCAGCAGTGCAGAG | AGTTGGCTGTCTGTGAAGTC | RT-qPCR |
| *Cdh2* | TATGCAAGACTGGATTTCCTGA | CTGGCTCGCTGCTTTCATAC | RT-qPCR |
| *Fermt2* | TTCGCAGCCTTGCAGTATCA | CTCCAGGTCAGAAAGGGCAG | RT-qPCR |
| *PECAM1* | GAACAGGACCGCGTTTTATCC | ATTCCGTCACGGTGACCAGTT | RT-qPCR |
| *CDH5* | ATGAGAATGACAATGCCCCG | TGTCTATTGCGGAGATCTGCAG | RT-qPCR |
| *NOS3* | TGATGGCGAAGCGAGTGAA | ACTCATCCATACACAGGACCCG | RT-qPCR |
| *VWF* | TGCGAAGTACCTTGGTTACCCA | TAATCGTCAGTACATGCCCCG | RT-qPCR |
| *KDR* | TGCGAAGTACCTTGGTTACCCA | TAATCGTCAGTACATGCCCCG | RT-qPCR |
| *ACTA2* | TGAGCGTGGCTATTCCTTCGT | GCAGTGGCCATCTCATTTTCAA | RT-qPCR |
| *TAGLN* | GGCTGAAGAATGGCGTGATT | TCTGCTTGAAGACCATGGAGG | RT-qPCR |
| *SMTN* | TGGAGTCCATGAACGATGTGG | TCAATCTCCTGAGCCCGTACAC | RT-qPCR |
| *CDH2* | AGGCTTCTGGTGAAATCGCA | TGCAGTTGCTAAACTTCACATTG | RT-qPCR |
| *DDR2* | TTTTTGGGTTGGGGAAACGC | AACCCCAGGCAACTTGTAGG | RT-qPCR |
| *S100A4* | TCTTGGTTTGATCCTGACTGCT | GCCCGAGTACTTGTGGAAGG | RT-qPCR |
| *SNAI1* | AATCCAGAGTTTACCTTCCAGCA | TCCCAGATGAGCATTGGCAG | RT-qPCR |
| *SNAI2* | TCGGACCCACACATTACCTTG | AAAAGGCTTCTCCCCCGTGT | RT-qPCR |
| *TWIST1* | TTCTCGGTCTGGAGGATGGA | AATGACATCTAGGTCTCCGGC | RT-qPCR |
| *TWIST2* | CTCAGCTACGCCTTCTCCG | CGACGGACAGCCCTGG | RT-qPCR |
| *FERMT2* | CGAGAATCTTGGAGGCCCAT | TTTTGCCCCCTTGGAACCTT | RT-qPCR |
| *YBX1* | AAGGAGAAAAGGGTGCGGAG | CCTACGACGTGGATAGCGTC | RT-qPCR |
| *SRF* | GGACAGTGCAGATCCCTGTTTC | TCCAGGTTCACCACCTGTAGCT | RT-qPCR |
| *miR-200c-3p* | CTGCCGGGTAATGATGGA | miRNA universal reverse primer (Invitrogen, A11193-051) | RT-qPCR |
| *miR-141-3p* | GCTAACACTGTCTGGTAAAGATGG | miRNA universal reverse primer (Invitrogen, A11193-051) | RT-qPCR |
| *miR-200a* | TAACACTGTCTGGTAACGATGT | miRNA universal reverse primer (Invitrogen, A11193-051) | RT-qPCR |
| *miR-200b* | TAATACTGCCTGGTAATGATGA | miRNA universal reverse primer (Invitrogen, A11193-051) | RT-qPCR |
| *miR-429* | TAATACTGTCTGGTAAAACCGT | miRNA universal reverse primer (Invitrogen, A11193-051) | RT-qPCR |
| *VWF* | CGGCAACTTTCAAGTCCTGC | GGTCAAGGTCCCTTCTTGGG | RT-qPCR/RIP |
| *VWF* (Intron-1) | CGATTGTTACCCTTGGGCCT | TCAGGGCATGCAGTAGCAAA | RT-qPCR/RIP |
| *CDH5* | ATGAGATCGTGGTGGAAGCG | TGTGTACTTGGTCTGGGTGAAG | RT-qPCR/RIP |
| *CDH5* (Intron-1) | CGGTGCCTTACCCTTACTGG | AGGCTCGGAGCTTCCTTTTC | RT-qPCR/RIP |
| pGL3-*ACTA2* | AAAGACCTCGAGGGTAGGCAAGTGGACTGAAGA | GAAACCAAGCTTAGGACAGTGAACCCATCCCTT | *ACTA2* gene promoter clone |
| pGL3-*ACTA2*-SRF^mut^ | GTAGTGTTGTATTCCTGTGCCAAGTACAAGGTAC | GTACCTTGTACTTGGCACAGGAATACAACACTAC | SRF binding site mutation |
| pGL3-*TAGLN* | CGTGGCCTCGAGGAAGACGCACTCGGGGCC | GACCTGAAGCTTGGGGCGCTGGCTGGGTGAG | *TAGLN* gene promoter clone |
| pGL3-*TAGLN*-SRF^mut^ | GGCAGGGTCCTGTTTCCTGTGAACTTTTCCCG | CGGGAAAGTTCACAGGAAACAGGACCCTGCC | SRF binding site-1 mutation |
| pGL3-*TAGLN*-SRF^mut^ | GTGTCTTTCCTTCCTGTGAAAGCCTGTGTGGAG | CTCCACACAGGCTTTCACAGGAAGGAAAGACAC | SRF binding site-2 mutation |
| pmiR-*FERMT2*-WT | ACCATGGAGCTCATAGGAATACTGTTTAATGAAACTC | TCTAGAACGCGTATTAAATAAAAGTTTTGCTTT | *FERMT2* 3**'**UTR reporter clone |
| pmiR-*FERMT2*-BS1^mut^ | CCATATTGTATTACTTTTCCGAGGCACCAGCATA | TATGCTGGAGCCTCGGAAAAGTAATACAATATGG | *miR-200c-3p* binding site 1 mutation |
| pmiR-*FERMT2*-BS2^mut^ | GAGAGGTGGATTACTGAGGCAGTTCAATAATCCATGG | CCATGGATTATTGAACTGCCTCAGTAATCCACCTCTC | *miR-200c-3p* binding site 2 mutation |
| *ACTA2* -P1 | CTACACACTAAGGCTATTGTAG | TTTTATATGTTGTACCTTGTAC | CHIP (*ACTA2* gene promoter region with CArG) |
| *ACTA2* -P2 | CTCACTTTGCTGGGCTGAGT | GGCCAGCCTTGTGAATTAGC | CHIP (*ACTA2* gene promoter region without CArG) |
| *TAGLN* -P1 | GGGTCCTGTCCATAAAAGGCT | TGACTCCACACAGGCTCCATA | CHIP (*TAGLN* gene promoter region with CArG) |
| *TAGLN* -P2 | CTGGTGTGGAGTAGGTCCTCAG | CTCCTCCTGGGGCTTTTAGCTC | CHIP (*TAGLN* gene promoter region without CArG) |
| Lenti-*miR-200c* | GTGCTGGTTAACGGTAAATCGGTGTGTGTCGC | CACCAGCTCGAGCCGACAGAGAACTACGGTGC | For generating human *miR-200c-3p* lentivirus |
